# Supplementary material for: Myeloid cell deficiency of p38γ/p38δ protects against candidiasis and regulates antifungal immunity
Source: EMBO Mol Med. 2018 Apr 16;10(5):e8485. doi: 10.15252/emmm.201708485 (PMC5938613; doi:10.15252/emmm.201708485)
Supplement: Supplementary file 1 — Appendix [file EMMM-10-e8485-s001.pdf]

Appendix

Myeloid cell deficiency of p38γ/p38δ protects against candidiasis and regulates antifungal immunity

Table of Contents:

1. Appendix Methods and References

2. Appendix Table S1. Information about the antibodies used in this study.

3. Appendix Table S2. Statistical analysis for main figures.

4. Appendix Table S3. Information about the statistical analysis performed for appendix figures.

5. Appendix Figures and Figure legends S1 to S8

Appendix Methods

Mouse treatments.

WT and p38γ/δ$^{-/-}$ mice were injected intraperitoneally (i.p) with 30 mg per kg body weight of ibuprofen (IBU, sodium salt, SIGMA) (or with the same volume of the vehicle PBS) and after 1 h infected intravenously with 1 x 10^5 CFU of *C. albicans*. 24 h post-infection mice were injected i.p. with 30 mg per kg body weight of IBU per day or with the same volume of the vehicle PBS for the indicated days. The same protocol was used in mice treated with the compound N-acetylcysteine (NAC) from SIGMA. The NAC dose used in these experiments was 200 mg per kg body weight per day of NAC or the same volume of the vehicle PBS for the days indicated in the figures. The survival curves shown in Figures S5C and 5I were performed simultaneously and the control groups (WT and p38γ/δ$^{-/-}$ mice infected with *C. albicans* alone without IBU or NAC treatment) are the same for both treatments.

Mice were injected i.p with 10 mg per kg body weight of BIRB796 or SB203580, or with the same volume of the vehicle DMSO, and after 1 h infected with 1 x 10^5 CFU of *C. albicans*. 24 h post-infection mice were injected i.p. with 10 mg per kg body weight per day of BIRB796 or SB203580, or with the same volume of the vehicle DMSO up to 3 days.

Flow cytometry analysis

Renal leukocyte infiltration was analysed by flow cytometry on cell suspensions from kidney homogenates that were digested with 0.2 mg/ml liberase (Roche) and 0.1 mg/ml DNase I (Roche) for 20 min at 37°C on a shaking platform and filtered through 40 µm cell strainers (Falcon). The flow cytometry analysis was performed with 1x10^6 cells per

condition; cells were stained with combinations of fluorescent-labelled antibodies against the cell surface markers CD45, CD4, CD8, Ly6G and F4/80 and analysed in a Cytomics FC500 flow cytometer (Beckman Coulter). Profiles were analysed with Kalooza software (Beckman Coulter); leukocytes were gated as $CD45^+$ cells.

Bone marrow-derived macrophages (BMDM) preparation and stimulation.

Bone marrow cells were allowed to differentiate on bacteria-grade plastic dishes in DMEM with 20% FBS and 30% L929 cell-conditioned media (CSF-1 source). After six days, adherent cells were collected, counted and plated in DMEM with 0.05% FBS at a constant density (1.5 x 10^6 cells/ml). At 12 h after re-plating, cells were stimulated with the different compounds. Cells were lysed in buffer A (50 mM Tris-HCl pH 7.5, 1 mM EGTA, 1 mM EDTA, 0.15 M NaCl, 1 mM sodium orthovanadate, 10 mM sodium fluoride, 50 mM sodium β-glycerophosphate, 5 mM pyrophosphate, 0.27 M sucrose, 0.1 mM phenylmethylsulphonyl fluoride, 1% (v/v) Triton X-100) plus 0.1% (v/v) 2-mercaptoethanol and complete proteinase inhibitor cocktail (Roche; East Sussex, UK). Lysates were centrifuged (15,500 x g, 15 min, 4°C) and supernatants removed, quick-frozen in liquid nitrogen, and stored at -80°C.

Neutrophils isolation

Neutrophils were obtained from adult mice blood. Mature neutrophils were isolated by gradient centrifugation over Histopaque 1119 (density, 1.119 g/ml) and Histopaque 1077 (density, 1.077 g/ml) according to the manufacturer's instructions at 400 x *g* for 30 min at 25°C, followed by hypotonic red blood cell lysis, Neutrophils recovered at the interface of the Histopaque 1119 and Histopaque 1077 layers were 80–90% pure and >95% viable as determined by flow cytometry. Neutrophils were washed twice and

were suspended in RPMI medium supplemented with 10% FBS.

Isolation and stimulation of human monocytes.

Venous blood was drawn from the cubital vein of four healthy volunteers into three 10 ml EDTA tubes, after obtaining informed consent. Blood was diluted 1:1 in PBS and the PBMCs were separated from the erythrocytes and granulocytes by centrifugation on a Ficoll gradient (G&E Healthcare). Cells were washed twice in PBS, suspended in RPMI 1640 medium and counted in a Beckman Coulter counter. Human monocytes were isolated from PBMC by centrifugation in a Percol (Sigma) gradient. Briefly, in a 15 ml tube, 10 ml of Percol diluted 1:1 in PBS were added to 2 $x10^8$ PBMCs suspended in 3 ml of RPMI 1640 culture medium. After centrifugation the monocytes in the interphase were removed, washed twice in cold PBS, suspended in RPMI 1640 and counted. Human monocytes were cultured in eppendorf tubes at a concentration of $1x10^6$/eppendorf and pre-incubated with DMSO, C34 or SB203580 at the indicated concentrations for 1 h at 37°C 5% CO_2, before 1h stimulation with 1 x 10^6/ml heat killed *C. albicans* (InvivoGen). After 24 h, the supernatants were collected and stored at -80°C until assayed. Informed consent was obtained from all healthy volunteers. The experiments conformed to the principles set out in the WMA Declaration of Helsinki and the Department of Health and Human Services Belmont Report.

Antibodies

Antibodies to total ERK1/2, active phosphorylated ERK1/2 (Thr202/Tyr204; P-ERK1/2), to total and active phosphorylated MKK1 (Ser217/221, P-MKK1), to total JNK1/2, phospho-p105 NFκB1 (Ser933; P-p105) and active phospho-p38MAPK (Thr180-Tyr182; P-p38) were purchased from Cell Signaling Technology. Anti-TPL2, -

β-actin and -p38α were from Santa Cruz, anti-active phospho-JNK1/2 (Thr183-Tyr185; P-JNK) from Biosource. Anti-p38γ and -p38δ antibodies for immunoprecipitation and immunoblotting were raised and purified as described (Cuenda, Cohen et al., 1997, Goedert, Cuenda et al., 1997) by the Division of Signal Transduction Therapy (DSTT), University of Dundee, Dundee, United Kingdom. Anti-CD4 was from Beckman Coulter; anti-CD45 and anti-CD8 were from Biolegend; anti-F4/80, and anti-Ly6G from Bioscience. The dilution of the antibodies used in this study is described in Appendix Table S1.

Immunoblot

Protein samples were resolved in SDS-PAGE and transferred to nitrocellulose membranes, blocked (30 min) in TBST buffer (50 mM Tris/HCl pH 7.5, 0.15 M NaCl, 0.1% (v/v) Tween) with 5% (w/v) dry milk, then incubated in TBST buffer with 5% (w/v) dry milk and 0.5-1 μg/ml antibody (2 h, room temperature (RT) or overnight, 4°C). Protein was detected using either horseradish peroxidase-conjugated secondary antibodies and the enhanced chemiluminescence reagent (Amersham Pharmacia Biotech), or fluorescently labelled secondary antibodies (Invitrogen) and the Odyssey infrared imaging system.

Gene expression analysis.

cDNA for real-time quantitative PCR (qPCR) was generated from 0.5 μg total RNA using the High Capacity cDNA Reverse Transcription Kit (Applied Biosystems) in a 10 μl final reaction volume. Real-time qPCR reactions were performed in triplicate using 3 μl/well of two serial dilutions (1/40) of each cDNA, 0.3 μM of each primer, and 1x Fluocycle SYBR Green Mix for real-time qPCR (Genycell-EuroClone) in a volume of 8

μl in MicroAmp Optical 384-well plates (Applied Biosystems). PCR reactions were carried out in an ABI PRISM 7900HT (Applied Biosystems) and SDS v2.2 software was used to analyze results by the Comparative Ct Method (ΔΔCt). X-fold change in mRNA expression was quantified relative to non-stimulated wild-type cells, and β-actin or GAPDH mRNA was used as control.

Cytokine measurement.

Cytokine concentrations in BMDM culture supernatant and mouse serum samples were measured using the Luminex-based MilliPlex Mouse cytokine/chemokine immunoassay and the Luminex-based Bio-Plex Mouse Grp I Cytokine 23-Plex Panel (Bio-Rad). Cytokine concentrations in human monocyte supernatant were determined by ELISA (R&D System and Bioscience) according to manufacturer's protocol.

Primer sequences used for quantitative real-time PCR.

IL-6, forward 5'-GAGGATACCACTCCCAACAGACC and reverse 5'-AAGTGCATCATGGTTGTTCATACA; *IL-10*, forward 5'-CAGGACTTTAAGGGTTACTTG and reverse 5'- ATTTTCACAGGGGAGAAATC; *IL1β*, forward 5'-TGGTGTGTGACGTTCCCATT and reverse 5'-CAGCACGAGGCTTTTTTGTTG; *TNFα*, forward 5'-CTGTAGCCCACGTCGTAGC and reverse 5'-TTGAGATCCATGCCGTTG; *IFNβ*, forward 5'-TCAGAATGAGTGGTGGTTGC and reverse 5'-GACCTTTCAAATGCAGTAGATTCA; *MIP-2 (CXCL2)*, forward 5'-CCTGGTTCAGAAAATCATCCA and reverse 5'- CTTCCGTTGAGGGACAGC; *CCL2*, forward 5'-TTGGGATCATCTTGCTGGTG and reverse 5'-TCTGGGCCTGCTGTTCACA; *KC*, forward 5'-CCTTGACCCTGAAGCTCCCT and

reverse 5'-CGGTGCCATCAGAGCAGTCT; *β-actin*, forward 5'-AAGGAGATTACTTGCTCTGGCTCCTA and reverse 5'-ACTCATCGTACTCCTGCTTGCTGAT;

GAPDH, forward 5'- CCCATCACCATCTTCCAGGA and reverse 5'-CGACATACTCAGCACCGGC; *iNOS* forward 5'-CAGCTGGGCTGTACAAACCTT and reverse 5'-CATTGGAAGTGAAGCGTTTCG.

References

Cuenda A, Cohen P, Buee-Scherrer V, Goedert M (1997) Activation of stress-activated protein kinase-3 (SAPK3) by cytokines and cellular stresses is mediated via SAPKK3 (MKK6); comparison of the specificities of SAPK3 and SAPK2 (RK/p38). The EMBO journal 16: 295-305

Goedert M, Cuenda A, Craxton M, Jakes R, Cohen P (1997) Activation of the novel stress-activated protein kinase SAPK4 by cytokines and cellular stresses is mediated by SKK3 (MKK6); comparison of its substrate specificity with that of other SAP kinases. The EMBO journal 16: 3563-71

Appendix Table S1. Information about the antibodies used in this study.

Antibody	Name	Provider	Method	Dilution
P-ERK1/2	Phospho-p44/42 MAPK (Erk1/2) (Thr202/Tyr204) Antibody	Cell Signaling	WB	1/2000
ERK1/2	p44/42 MAPK (Erk1/2) Antibody	Cell Signaling	WB	1/1000
P-MKK1	Phospho-MEK1/2 (Ser217/221) Antibody	Cell Signaling	WB	1/500
MKK1	MEK1/2 Antibody	Cell Signaling	WB	1/500
Tpl2	Cot (M-20)	Santa Cruz Biotechnology, Inc.	WB	1/1000
P-p38	Phospho-p38 MAP Kinase (Thr180/Tyr182) Antibody	Cell Signaling	WB	1/1000
p38α	p38α (C-20)	Santa Cruz Biotechnology, Inc.	WB	1/1000
p38γ	p38γ (GST-SAPK3) S524A 1st Bleed	DSTT*	WB	1/1000
p38δ	p38δ (GST-SAPK4) S526A 3rd Bleed	DSTT*	WB	1/1000
P-JNK1/2	Rabbit (polyclonal) Anti-JNK1&2 [pTpY183/185] Phosphospecific Antibody, Unconjugated	Biosurce	WB	1/1000
JNK1/2	SAPK/JNK Rabbit Ab	Cell Signaling	WB	1/1000
P-p105	Phospho-NF-κB p105 (Ser933) (18E6) Rabbit mAb	Cell Signaling	WB	1/1000
p105	NF-κB1 p105 Antibody	Cell Signaling	WB	1/1000
p38γ	p38γ (SAPK3 C-Terminal) [KPPRNLGARVPKETAL]	DSTT*	IP	2 μg/IP
p38δ	p38δ (GST-SAPK4) S526A 3rd Bleed	DSTT*	IP	5 μg/IP
CD45	CD45 Monoclonal Antibody (30-F11), APC #17-0451-82	eBioscience™	FAC	1/100
F4/80	F4/80 Monoclonal Antibody (BM8), Biotin #13-4801-85	eBioscience™	FAC	1/200
Streptavidin	ECD (PE-CF594) Streptavidin	BD Bioscience	FAC	1/100
CD4	RAT ANTI-MOUSE CD4/L3T4-FITC (CLONE GK1.5) #731999	Beckman Coulter	FAC	1/100
CD8	PE/Cy7 anti-mouse CD8a Antibody #100712	Biolegend	FAC	1/200
Ly6G	PE Rat Anti-Mouse Ly-6G Clone 1A8 (RUO) #551461	BD Bioscience	FAC	1/50

* DSTT (Dundee): Division of Signal Transduction Therapy; University of Dundee (Dundee, UK)

Appendix Table S2. Statistical analysis for main figures.

Figure	Panel	Experiment	*n*-value	*p*-value	Significance
1	A	TNF WT vs p38γ/δ-/- (time 0)	3	0.646	ns
		TNF WT vs p38γ/δ-/- (time 1)	3	0.658	ns
		TNF WT vs p38γ/δ-/- (time 2)	3	0.346	ns
		TNF WT vs p38γ/δ-/- (time 4)	3	0.559	ns
		TNF WT vs p38γ/δ-/- (time 6)	3	0.472	ns
		IL6 WT vs p38γ/δ-/- (time 0)	3	0.694	ns
		IL6 WT vs p38γ/δ-/- (time 1)	3	0.521	ns
		IL6 WT vs p38γ/δ-/- (time 2)	3	0.689	ns
		IL6 WT vs p38γ/δ-/- (time 4)	3	0.389	ns
		IL6 WT vs p38γ/δ-/- (time 6)	3	0.385	ns
		IL1β WT vs p38γ/δ-/- (time 0)	3	0.413	ns
		IL1β WT vs p38γ/δ-/- (time 1)	3	0.040	*
		IL1β WT vs p38γ/δ-/- (time 2)	3	0,124	ns
		IL1β WT vs p38γ/δ-/- (time 4)	3	0.409	ns
		IL1β WT vs p38γ/δ-/- (time 6)	3	0.953	ns
		IL10 WT vs p38γ/δ-/- (time 0)	3	0.540	ns
		IL10 WT vs p38γ/δ-/- (time 1)	3	0.015	*
		IL10 WT vs p38γ/δ-/- (time 2)	3	0.383	ns
		IL10 WT vs p38γ/δ-/- (time 4)	3	0.412	ns
		IL10 WT vs p38γ/δ-/- (time 6)	3	0.035	*
		KC WT vs p38γ/δ-/- (time 0)	3	0.184	ns
		KC WT vs p38γ/δ-/- (time 1)	3	0.080	ns
		KC WT vs p38γ/δ-/- (time 2)	3	0.340	ns
		KC WT vs p38γ/δ-/- (time 4)	3	0.036	*
		KC WT vs p38γ/δ-/- (time 6)	3	0.239	ns
		MIP2 WT vs p38γ/δ-/- (time 0)	3	0.193	ns
		MIP2 WT vs p38γ/δ-/- (time 1)	3	0.0267	*
		MIP2 WT vs p38γ/δ-/- (time 2)	3	0.0492	*
		MIP2 WT vs p38γ/δ-/- (time 4)	3	0.0355	*
		MIP2 WT vs p38γ/δ-/- (time 6)	3	0.0136	*
		CCL2 WT vs p38γ/δ-/- (time 0)	3	0.966	ns
		CCL2 WT vs p38γ/δ-/- (time 1)	3	0.048	*
		CCL2 WT vs p38γ/δ-/- (time 2)	3	0.711	ns
		CCL2 WT vs p38γ/δ-/- (time 4)	3	0.435	ns
		CCL2 WT vs p38γ/δ-/- (time 6)	3	0.064	ns
	D	Ctr WT vs Ctr p38γ/δ-/-<br>Curdlan WT vs Curdlan p38γ/δ-/-	3<br>3	0.121<br>0.046	ns<br>*
2	A	Ctr TPL2+/+ vs Ctr TPL2-/-	6	0.703	ns
		LPS TPL2+/+ vs LPS TPL2-/-	6	< 0.0001	***
		Curd TPL2+/+ vs Curd TPL2-/-	6	< 0.0001	***
		HKCa TPL2+/+ vs HKCa TPL2-/-	6	0.0009	***
		Zym TPL2+/+ vs Zym TPL2-/-	6	0.0002	***
	C	WT Curd vs Curd+C34<br>p38γ/δ-/- Curd vs Curd+C34<br>Curd WT vs p38γ/δ-/-<br>Curd+C34 WT vs p38γ/δ-/-	3<br>3<br>3<br>3	0.018<br>0.167<br>0.022<br>0.423	*<br>ns<br>*<br>ns
3	A	WT vs p38γ/δ-/-	20	0.0005	***
		WT vs LysMp38γ/δ-/-	20	0.0008	***
		p38γ/δ-/- vs LysMp38γ/δ-/-	20	0.111	ns

	B	WT vs p38γ/δ-/-	13; 8	0.027	*
		WT vs LysMp38γ/δ-/-	13: 9	0.013	*
		p38γ/δ-/- vs LysMp38γ/δ-/-	8; 9	0.774	ns
	D	TPL2+/+ vs TPL2-/-	12	0.896	ns
	E	TPL2+/+ vs TPL2-/-	4	0.0226	*
4	A	TNF WT vs p38γ/δ-/- (Ctr)	3	0.419	ns
		TNF WT vs LysMp38γ/δ-/- (Ctr)	3	0.596	ns
		TNF p38γ/δ-/- vs LysMp38γ/δ-/- (Ctr)	3	0.189	ns
		TNF WT vs p38γ/δ-/- (Day1)	5	0.020	*
		TNF WT vs LysMp38γ/δ-/- (Day1)	5	0.0021	**
		TNF p38γ/δ-/- vs LysMp38γ/δ-/- (Day1)	5	0.032	*
		TNF WT vs p38γ/δ-/- (Day3)	5; 4	0.419	ns
		TNF WT vs LysMp38γ/δ-/- (Day3)	5; 3	0.596	ns
		TNF p38γ/δ-/- vs LysMp38γ/δ-/- (Day3)	4; 3	0.292	ns
		IL6 WT vs p38γ/δ-/- (Ctr)	3	0.6388	ns
		IL6 WT vs LysMp38γ/δ-/- (Ctr)	3	0.6705	ns
		IL6 p38γ/δ-/- vs LysMp38γ/δ-/- (Ctr)	3	0.8989	ns
		IL6 WT vs p38γ/δ-/- (Day1)	5	0.0029	**
		IL6 WT vs LysMp38γ/δ-/- (Day1)	5	0.0045	**
		IL6 p38γ/δ-/- vs LysMp38γ/δ-/- (Day1)	5	0.0411	*
		IL6 WT vs p38γ/δ-/- (Day3)	3	0.9745	ns
		IL6 WT vs LysMp38γ/δ-/- (Day3)	3	0.4580	ns
		IL6 p38γ/δ-/- vs LysMp38γ/δ-/- (Day3)	3	0.6122	ns
		IL1β WT vs p38γ/δ-/- (Ctr)	3	0.5138	ns
		IL1β WT vs LysMp38γ/δ-/- (Ctr)	3	0.0580	ns
		IL1β p38γ/δ-/- vs LysMp38γ/δ-/- (Ctr)	3	0.4135	ns
		IL1β WT vs p38γ/δ-/- (Day1)	7; 8	0.0282	*
		IL1β WT vs LysMp38γ/δ-/- (Day1)	7; 4	0.0252	*
		IL1β p38γ/δ-/- vs LysMp38γ/δ-/- (Day1)	8; 4	0.5005	ns
		IL1β WT vs p38γ/δ-/- (Day3)	4	0.9188	ns
		IL1β WT vs LysMp38γ/δ-/- (Day3)	4	0.1303	ns
		IL1β p38γ/δ-/- vs LysMp38γ/δ-/- (Day3)	4	0.0713	ns
	B	CD45 WT vs p38γ/δ-/- (Day0)	9; 14	0.733	ns
		CD45 WT vs LysMp38γ/δ-/- (Day0)	9; 7	0.020	*
		CD45 p38γ/δ-/- vs LysMp38γ/δ-/- (Day0)	14; 7	0.025	*
		CD45 WT vs p38γ/δ-/- (Day1)	9; 13	0.383	ns
		CD45 WT vs LysMp38γ/δ-/- (Day1)	9; 7	0.008	**
		CD45 p38γ/δ-/- vs LysMp38γ/δ-/- (Day1)	13; 7	0.107	ns
		CD45 WT vs p38γ/δ-/- (Day3)	8; 7	0.001	*
		CD45 WT vs LysMp38γ/δ-/- (Day3)	8; 7	0.0002	**
		CD45 p38γ/δ-/- vs LysMp38γ/δ-/- (Day3)	7	0.107	ns
		Ly6G WT vs p38γ/δ-/- (Day0)	9; 10	0.443	ns
		Ly6G WT vs LysMp38γ/δ-/- (Day0)	9; 8	0.139	ns
		Ly6G p38γ/δ-/- vs LysMp38γ/δ-/- (Day0)	10; 8	0.122	ns
		Ly6G WT vs p38γ/δ-/- (Day1)	15; 11	0.002	**

		Ly6G WT vs LysMp38γ/δ-/- (Day1)	15; 6	0.002	**
		Ly6G p38γ/δ-/- vs LysMp38γ/δ-/- (Day1)	11; 6	0.435	ns
		Ly6G WT vs p38γ/δ-/- (Day3)	5	0.0001	***
		Ly6G WT vs LysMp38γ/δ-/- (Day3)	5	< 0.0001	***
		Ly6G p38γ/δ-/- vs LysMp38γ/δ-/- (Day3)	5	0.013	*
		F4/80 WT vs p38γ/δ-/- (Day0)	9; 10	0.407	ns
		F4/80 WT vs LysMp38γ/δ-/- (Day0)	9; 8	0.026	*
		F4/80 p38γ/δ-/- vs LysMp38γ/δ-/- (Day0)	10; 8	0.128	ns
		F4/80 WT vs p38γ/δ-/- (Day1)	14; 13	0.526	ns
		F4/80 WT vs LysMp38γ/δ-/- (Day1)	14; 6	0.004	**
		F4/80 p38γ/δ-/- vs LysMp38γ/δ-/- (Day1)	13; 6	0.084	ns
		F4/80 WT vs p38γ/δ-/- (Day3)	6; 5	0.048	*
		F4/80 WT vs LysMp38γ/δ-/- (Day3)	6; 5	0.009	**
		F4/80 p38γ/δ-/- vs LysMp38γ/δ-/- (Day3)	5	0.043	*
	C	F4/80 WT vs p38γ/δ-/-	4	0.9111	ns
		F4/80 WT vs LysMp38γ/δ-/-	4	0.0015	**
		F4/80 p38γ/δ-/- vs LysMp38γ/δ-/-	4	0.0318	*
		Ly6G WT vs p38γ/δ-/-	4	< 0.0001	***
		Ly6G WT vs LysMp38γ/δ-/-	4	< 0.0001	***
		Ly6G p38γ/δ-/- vs LysMp38γ/δ-/-	4	0.0332	*
	D	MIP-2 WT vs p38γ/δ-/- (Day0)	3	0.515	ns
		MIP-2 WT vs LysMp38γ/δ-/- (Day0)	3	0.412	ns
		MIP-2 p38γ/δ-/- vs LysMp38γ/δ-/- (Day0)	3	0.439	ns
		MIP-2 WT vs p38γ/δ-/- (Day1)	4	0.026	*
		MIP-2 WT vs LysMp38γ/δ-/- (Day1)	4	0.017	*
		MIP-2 p38γ/δ-/- vs LysMp38γ/δ-/- (Day1)	4	0.116	ns
		KC WT vs p38γ/δ-/- (Day0)	3	0.455	ns
		KC WT vs LysMp38γ/δ-/- (Day0)	3	0.409	ns
		KC p38γ/δ-/- vs LysMp38γ/δ-/- (Day0)	3	0.329	ns
		KC WT vs p38γ/δ-/- (Day1)	5	0.0401	*
		KC WT vs LysMp38γ/δ-/- (Day1)	5	0.0065	*
		KC p38γ/δ-/- vs LysMp38γ/δ-/- (Day1)	5	0.4320	ns
		CCL2 WT vs p38γ/δ-/- (Day0)	3	0.312	ns
		CCL2 WT vs LysMp38γ/δ-/- (Day0)	3	0.142	ns
		CCL2 p38γ/δ-/- vs LysMp38γ/δ-/- (Day0)	3	0.674	ns
		CCL2 WT vs p38γ/δ-/- (Day1)	4	0.027	*
		CCL2 WT vs LysMp38γ/δ-/- (Day1)	4	0.0013	**
		CCL2 p38γ/δ-/- vs LysMp38γ/δ-/- (Day1)	4	0.056	ns
5	A	iNOS WT vs p38γ/δ-/- (time 0)	3	0.0588	ns
		iNOS WT vs p38γ/δ-/- (time 1)	3	0.247	ns
		iNOS WT vs p38γ/δ-/- (time 2)	3	0.0296	*
		iNOS WT vs p38γ/δ-/- (time 4)	3	0.0056	***
	C	WT vs p38γ/δ-/-	7	< 0.0001	***
	E	WT vs p38γ/δ-/-	4	0.0379	*
	H	WT vs p38γ/δ-/- (Day 0)	3	0.905	ns

		WT vs p38γ/δ-/- (Day 3)	5	0.033	*
	I	WT NAC vs WT CA	10; 8	0.0021	**
		WT CA vs WT CA+NAC	8	0.022	*
		p38γ/δ-/- NAC vs p38γ/δ-/- CA	10; 8	0.154	ns
		p38γ/δ-/- CA vs p38γ/δ-/- CA+NAC	8	0.003	**
		WT CA vs p38γ/δ-/- CA	8	0.026	*
		WT CA+NAC vs p38γ/δ-/- CA+NAC	8	0.070	ns
6	A	DMSO vs BIRB	5	0.022	*
		DMSO vs SB	5	0.672	ns
		BIRB vs SB	5	0.011	*
	B	DMSO vs BIRB	5	0.036	*
		DMSO vs SB	5	0.503	ns
		BIRB vs SB	5	0.064	ns
	C	DMSO vs BIRB	6	< 0.0001	***
		DMSO vs SB	6	< 0.0001	***
		BIRB vs SB	6	0.527	ns
	D	DMSO vs BIRB	4	0.0089	**
		DMSO vs SB	4	0.3025	ns
		BIRB vs SB	4	0.0507	*

Appendix Table S3. Information about the statistical analysis performed for appendix figures S1 to S8.

Figure	Panel	Experiment	*n*-value	*p*-value	Significance
S1	D	Ctr WT vs MyD88-/-	4	0.169	ns
		LPS WT vs MyD88-/-	4	0.003	**
		Curd WT vs MyD88-/-	4	0.882	ns
		HKCa WT vs MyD88-/-	4	0.0031	**
	E	Ctr WT vs Dectin1-/-	4	0.902	ns
		LPS WT vs Dectin1-/-	4	0.373	ns
		Curd WT vs Dectin1-/-	4	0.029	*
		HKCa WT vs Dectin1-/-	4	0.119	*
	F	Ctr (-)PRT vs (+)PRT	4	0.840	ns
		LPS (-)PRT vs (+)PRT	4	0.393	ns
		Curd (-)PRT vs (+)PRT	4	0.021	*
S2	C	WT HKCa vs HKCa+C34 (1h)	3	0.0162	*
		p38γ/δ-/- HKCa vs HKCa+C34 (1h)	3	0.1220	ns
		WT HKCa vs p38γ/δ-/- HKCa (1h)	3	0.0152	*
		WT HKCa vs HKCa+C34 (3h)	3	0.0500	*
		p38γ/δ-/- HKCa vs HKCa+C34 (3h)	3	0.420	ns
		WT HKCa vs p38γ/δ-/- HKCa (3h)	3	0.0366	*
	E	TNF: HKCa vs HKCa+C34 (1)	4	0.9844	ns
		TNF: HKCa vs HKCa+C34 (2)	4	0.0500	*
		TNF: HKCa vs HKCa+C34 (3)	4	0.0001	**
		TNF: HKCa vs HKCa+SB (1)	4	0.133	ns
		TNF: HKCa vs HKCa+SB (2)	4	0.261	ns
		TNF: HKCa vs HKCa+SB (3)	4	0.619	ns
		IL6: HKCa vs HKCa+C34 (1)	4	0.860	ns
		IL6: HKCa vs HKCa+C34 (2)	4	0.322	ns
		IL6: HKCa vs HKCa+C34 (3)	4	< 0.0001	***
		IL6: HKCa vs HKCa+SB (1)	4	0.003	**
		IL6: HKCa vs HKCa+SB (2)	4	0.033	*
		IL6: HKCa vs HKCa+SB (3)	4	0.007	**
		IL10: HKCa vs HKCa+C34 (1)	4	0.0218	*
		IL10: HKCa vs HKCa+C34 (2)	4	0.0217	*
		IL10: HKCa vs HKCa+C34 (3)	4	< 0.0001	***
		IL10: HKCa vs HKCa+SB (1)	4	0.0188	*
		IL10: HKCa vs HKCa+SB (2)	4	0.084	ns
		IL10: HKCa vs HKCa+SB (3)	4	0.010	**
S3		Blood: WT vs p38γ/δ-/- (0.5)	6	0.057	ns
		Blood: WT vs LysMp38γ/δ-/- (0.5)	6	0.405	ns
		Blood: p38γ/δ-/- vs LysMp38γ/δ-/- (0.5)	6	0.053	ns
		Blood: WT vs p38γ/δ-/- (1)	6	0.014	*
		Blood: WT vs LysMp38γ/δ-/- (1)	6	0.011	*
		Blood: p38γ/δ-/- vs LysMp38γ/δ-/- (1)	6	0.180	ns
		Blood: WT vs p38γ/δ-/- (3)	6	0.117	ns
		Blood: WT vs LysMp38γ/δ-/- (3)	6	0.233	ns
		Blood: p38γ/δ-/- vs LysMp38γ/δ-/- (3)	6	0.209	ns
		Blood: WT vs p38γ/δ-/- (6.5)	6	0.015	*
		Blood: WT vs LysMp38γ/δ-/- (6.5)	6	0.392	ns
		Blood: p38γ/δ-/- vs LysMp38γ/δ-/-	6	0.105	ns

		(6.5)			
		Blood: WT vs p38γ/δ-/- (24)	6	0.5	ns
		Blood: WT vs LysMp38γ/δ-/- (24)	6	0.186	ns
		Blood: p38γ/δ-/- vs LysMp38γ/δ-/- (24)	6	0.187	ns
		Kidney: WT vs p38γ/δ-/- (6.5)	6	0.204	ns
		Kidney: WT vs LysMp38γ/δ-/- (6.5)	6	0.030	*
		Kidney: p38γ/δ-/- vs LysMp38γ/δ-/- (6.5)	6	0.0008	***
		Kidney: WT vs p38γ/δ-/- (24)	6	0.323	ns
		Kidney: WT vs LysMp38γ/δ-/- (24)	6	0.059	ns
		Kidney: p38γ/δ-/- vs LysMp38γ/δ-/- (24)	6	0.102	ns
		Kidney: WT vs p38γ/δ-/- (48)	6	0.190	ns
		Kidney: WT vs LysMp38γ/δ-/- (48)	6	0.200	ns
		Kidney: p38γ/δ-/- vs LysMp38γ/δ-/- (48)	6	0.355	ns
		Kidney: WT vs p38γ/δ-/- (72)	6	0.046	*
		Kidney: WT vs LysMp38γ/δ-/- (72)	6	0.042	*
		Kidney: p38γ/δ-/- vs LysMp38γ/δ-/- (72)	6	0.053	ns
		Spleen: WT vs p38γ/δ-/- (6.5)	3	0.013	*
		Spleen: WT vs LysMp38γ/δ-/- (6.5)	3	0.423	ns
		Spleen: p38γ/δ-/- vs LysMp38γ/δ-/- (6.5)	3	0.095	ns
		Spleen: WT vs p38γ/δ-/- (24)	3	0.491	ns
		Spleen: WT vs LysMp38γ/δ-/- (24)	3	0.393	ns
		Spleen: p38γ/δ-/- vs LysMp38γ/δ-/- (24)	3	0.340	ns
		Spleen: WT vs p38γ/δ-/- (48)	3	0.172	ns
		Spleen: WT vs LysMp38γ/δ-/- (48)	3	0.155	ns
		Kidney: p38γ/δ-/- vs LysMp38γ/δ-/- (48)	3	0.344	ns
		Spleen: WT vs p38γ/δ-/- (72)	3	0.193	ns
		Spleen: WT vs LysMp38γ/δ-/- (72)	3	0.164	ns
		Spleen: p38γ/δ-/- vs LysMp38γ/δ-/- (72)	3	0.048	*
		Liver: WT vs p38γ/δ-/- (6.5)	3	0.059	ns
		Liver: WT vs LysMp38γ/δ-/- (6.5)	3	0.0001	***
		Liver: p38γ/δ-/- vs LysMp38γ/δ-/- (6.5)	3	0.380	ns
		Liver: WT vs p38γ/δ-/- (24)	3	0.176	ns
		Liver: WT vs LysMp38γ/δ-/- (24)	3	0.435	ns
		Liver: p38γ/δ-/- vs LysMp38γ/δ-/- (24)	3	0.174	ns
		Liver: WT vs p38γ/δ-/- (48)	3	0.194	ns
		Liver: WT vs LysMp38γ/δ-/- (48)	3	0.261	ns
		Liver: p38γ/δ-/- vs LysMp38γ/δ-/- (48)	3	0.342	ns
		Liver: WT vs p38γ/δ-/- (72)	3	0.003	**
		Liver: WT vs LysMp38γ/δ-/- (72)	3	0.347	ns
		Liver: p38γ/δ-/- vs LysMp38γ/δ-/- (72)	3	0.079	ns
		Brain: WT vs p38γ/δ-/- (6.5)	3	0.013	*
		Brain: WT vs LysMp38γ/δ-/- (6.5)	3	0.407	ns
		Brain: p38γ/δ-/- vs LysMp38γ/δ-/- (6.5)	3	0.086	ns
		Brain: WT vs p38γ/δ-/- (24)	3	0.477	ns
		Brain: WT vs LysMp38γ/δ-/- (24)	3	0.263	ns

		Brain: p38γ/δ-/- vs LysMp38γ/δ-/- (24)	3	0.205	ns
		Brain: WT vs p38γ/δ-/- (48)	3	0.176	ns
		Brain: WT vs LysMp38γ/δ-/- (48)	3	0.196	ns
		Brain: p38γ/δ-/- vs LysMp38γ/δ-/- (48)	3	0.457	ns
		Brain: WT vs p38γ/δ-/- (72)	3	0.194	ns
		Brain: WT vs LysMp38γ/δ-/- (72)	3	0.165	ns
		Brain: p38γ/δ-/- vs LysMp38γ/δ-/- (72)	3	0.061	ns
S4	A	IFNγ WT vs p38γ/δ-/- (Day0)	4	0.086	ns
		IFNγ WT vs p38γ/δ-/- (Day1)	5	0.017	*
		IFNγ WT vs p38γ/δ-/- (Day3)	5	0.373	ns
		TNF WT vs p38γ/δ-/- (Day0)	3	0.059	ns
		TNF WT vs p38γ/δ-/- (Day1)	4	0.227	ns
		TNF WT vs p38γ/δ-/- (Day3)	4	0.0068	**
		IL1β WT vs p38γ/δ-/- (Day0)	4	0.077	ns
		IL1β WT vs p38γ/δ-/- (Day1)	5	0.306	ns
		IL1β WT vs p38γ/δ-/- (Day3)	5	0.141	ns
	B	TNF TPL2+/+ vs TPL2-/- (day 0)	3	0.131	ns
		TNF TPL2+/+ vs TPL2-/- (day 1)	3; 5	0.393	ns
		TNF TPL2+/+ vs TPL2-/- (day 3)	5	0.025	*
		IL6 TPL2+/+ vs TPL2-/- (day 0)	3	0.328	ns
		IL6 TPL2+/+ vs TPL2-/- (day 1)	5	0.0033	**
		IL6 TPL2+/+ vs TPL2-/- (day 3)	5; 7	0.568	ns
		IL1β TPL2+/+ vs TPL2-/- (day 0)	3	0.058	ns
		IL1β TPL2+/+ vs TPL2-/- (day 1)	5	0.0347	*
		IL1β TPL2+/+ vs TPL2-/- (day 3)	4; 6	0.00015	***
	C	CD4 WT vs p38γ/δ-/- (Day0)	10; 11	0.975	ns
		CD4 WT vs LysMp38γ/δ-/- (Day0)	10; 8	0.388	ns
		CD4 p38γ/δ-/- vs LysMp38γ/δ-/- (Day0)	11; 8	0.483	ns
		CD4 WT vs p38γ/δ-/- (Day1)	15; 13	0.983	ns
		CD4 WT vs LysMp38γ/δ-/- (Day1)	15; 6	0.020	*
		CD4 p38γ/δ-/- vs LysMp38γ/δ-/- (Day1)	13; 6	0.099	ns
		CD4 WT vs p38γ/δ-/- (Day3)	6; 5	0.633	ns
		CD4 WT vs LysMp38γ/δ-/- (Day3)	6; 5	0.155	ns
		CD4 p38γ/δ-/- vs LysMp38γ/δ-/- (Day3)	5	0.332	ns
		CD8 WT vs p38γ/δ-/- (Day0)	8; 10	0.164	ns
		CD8 WT vs LysMp38γ/δ-/- (Day0)	8	0.154	*
		CD8 p38γ/δ-/- vs LysMp38γ/δ-/- (Day0)	10; 8	0.110	*
		CD8 WT vs p38γ/δ-/- (Day1)	15; 13	0.145	ns
		CD8 WT vs LysMp38γ/δ-/- (Day1)	15; 6	0.564	ns
		CD8 p38γ/δ-/- vs LysMp38γ/δ-/- (Day1)	13; 6	0.216	ns
		CD8 WT vs p38γ/δ-/- (Day3)	6; 5	0.091	ns
		CD8 WT vs LysMp38γ/δ-/- (Day3)	6; 5	0.622	ns
		CD8 p38γ/δ-/- vs LysMp38γ/δ-/- (Day3)	5	0.134	ns
S5	A	WT vs WT+IBU	6; 4	0.167	ns
		WT vs p38γ/δ-/-	6; 8	0.044	*
		WT+IBU vs p38γ/δ-/-	4; 8	0.471	ns
		p38γ/δ-/- vs p38γ/δ-/-+IBU	8; 4	0.018	*
	B	WT vs WT+IBU	6	0.0057	**

		WT vs p38γ/δ-/-	6; 4	0.0046	**
		WT+IBU vs p38γ/δ-/-	6; 4	0.1117	ns
		p38γ/δ-/- vs p38γ/δ-/-+IBU	4	0.8062	ns
	C	WT+IBU vs WT+CA	6; 8	0.013	*
		WT+CA vs WT+CA+IBU	8	0.019	*
		p38γ/δ-/- +IBU vs p38γ/δ-/- +CA	6; 7	0.048	*
		p38γ/δ-/- +CA vs p38γ/δ-/- +CA+IBU	7	0.550	ns
		WT+CA vs p38γ/δ-/- +CA	8; 7	0.026	*
		WT+CA+IBU vs p38γ/δ-/- +CA+IBU	8; 7	0.023	*
S6	A	WT vs p38γ/δ-/-	6	0.848	ns
	C	Crt vs NAC	4	0.020	*
S7	A	DMSO vs BIRB (Day 1)	5	0.022	*
		DMSO vs SB (Day 1)	5	0.0012	**
		BIRB vs SB (Day 1)	5	0.094	ns
		DMSO vs BIRB (Day 3)	5; 4	0.068	ns
		DMSO vs SB (Day 3)	5	0.484	ns
		BIRB vs SB (Day 3)	4; 5	0.153	ns
	C	p38γ DMSO vs (+) BIRB	4	0.0248	*
		p38γ DMSO vs (+) SB	4	0.988	ns
		p38δ DMSO vs (+) BIRB	4	0.022	*
		p38δ DMSO vs (+) SB	4	0.371	ns
	D	DMSO vs BIRB	5; 4	0.879	ns
		DMSO vs SB	5	0.502	ns
		BIRB vs SB	4; 5	0.441	ns
	E	WT vs WT+BIRB	4	0.0354	*
		WT vs p38γ/δ-/-	4	0.0376	*
		WT+BIRB vs p38γ/δ-/-	4	0.1958	ns
		p38γ/δ-/- vs p38γ/δ-/-+BIRB	4	0.2085	ns
S8		Ly6G: WT vs p38γ/δflox/flox	4	0.145	ns
		Ly6G: WT vs LysM-Cre	4	0.874	ns
		Ly6G: p38γ/δflox/flox vs LysM-Cre	4	0.200	ns
		F4/80: WT vs p38γ/δflox/flox	4	0.693	ns
		F4/80: WT vs LysM-Cre	4	0.453	ns
		F4/80: p38γ/δflox/flox vs LysM-Cre	4	0.136	ns

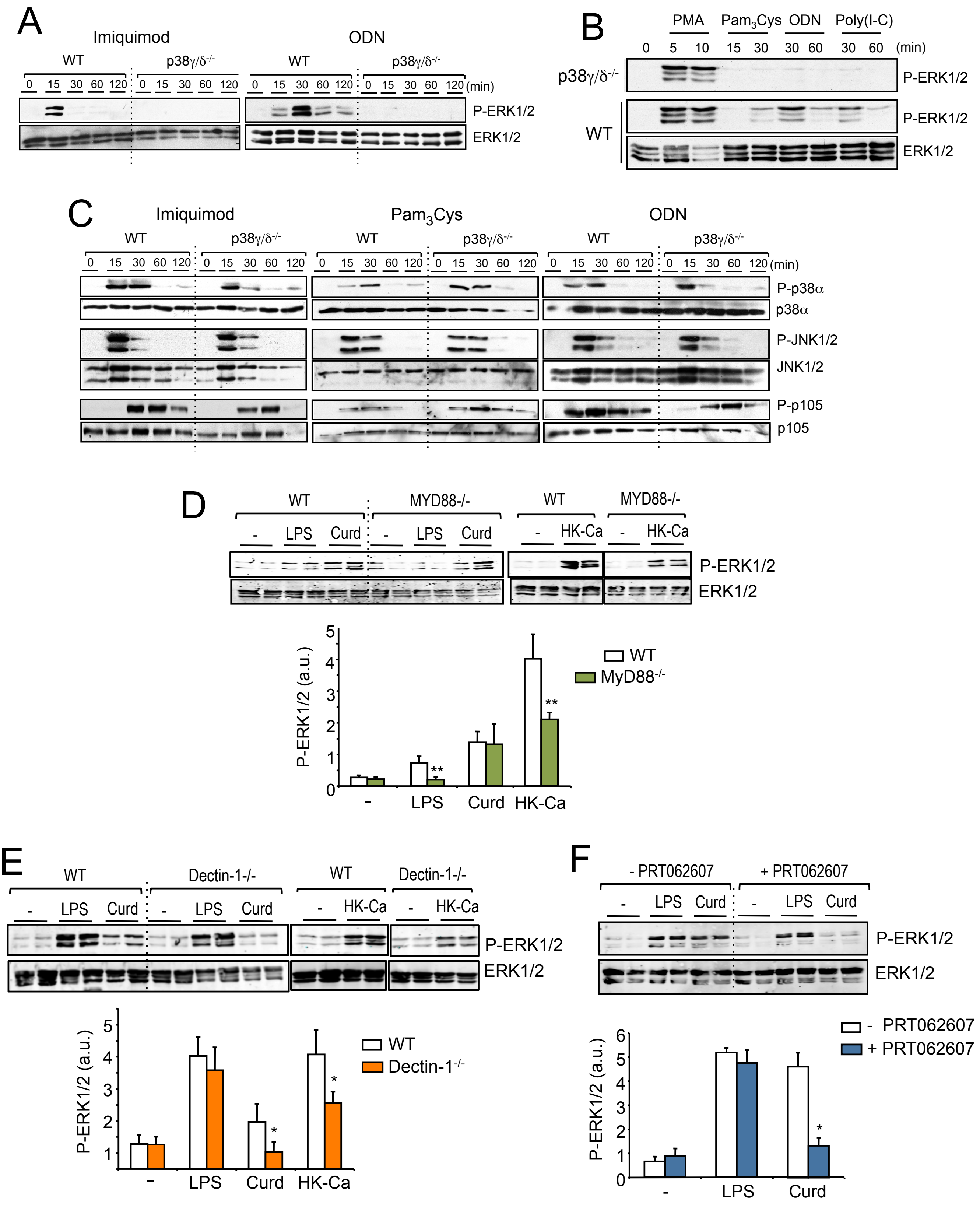
A
Imiquimod
ODN
WT
p38γ/δ-/-
0 15 30 60 120 (min)
P-ERK1/2
ERK1/2
B
PMA
Pam3Cys
ODN
Poly(I-C)
0 5 10 15 30 30 60 30 60 (min)
p38γ/δ-/-
WT
C
Pam3Cys
P-p38α
p38α
P-JNK1/2
JNK1/2
P-p105
p105
D
MYD88-/-
-
LPS
Curd
HK-Ca
P-ERK1/2 (a.u.)
MyD88-/-
**
E
Dectin-1-/-
*
F
- PRT062607
+ PRT062607

Figure S1

Fig S1. Effect of p38γ/p38δ deletion on ERK1/2 activation in BMDM.

(**A, B, C**) BMDM from WT or p38γ/δ$^{-/-}$ mice were stimulated with 5 μg/ml of Imiquimod, 100 ng/ml PMA, 200 ng/ml Pam3Cys, 250 ng/ml ODN or 1 μg/ml Poly (I-C) for the indicated times. Cell lysates were immunoblotted with the indicated antibodies. Representative immunoblots from two independent experiments are shown.

(D) BMDM from WT or MyD88$^{-/-}$ mice and **(E)** BMDM from WT or Dectin-1$^{-/-}$ mice were stimulated with 1 x 10^6 CFU/ml HK-Ca or 10 μg/ml Curdlan for 1 h, or with 100 ng/ml LPS for 15 min. Cell lysates were immunoblotted with the antibodies indicated (top). Representative blots from two independent experiments are shown. Bands were quantified using the Odyssey infrared imaging system (bottom) and data show mean ± SEM from two experiments in duplicate. *$p \leq 0.05$, **$p \leq 0.01$ relative to WT cells, in each condition.

(F) WT BMDM were incubated for 1 h in the absence (DMSO) or the presence of 10 μM PRT062607 and then stimulated with Curdlan as in (D), or with 100 ng/ml LPS for 30 min. Cell lysates were immunoblotted with the indicated antibodies (top). Representative blots from three independent experiments are shown. Bands were quantified as in (D, bottom), and data show mean ± SEM from two experiments in duplicate. **$p \leq 0.01$.

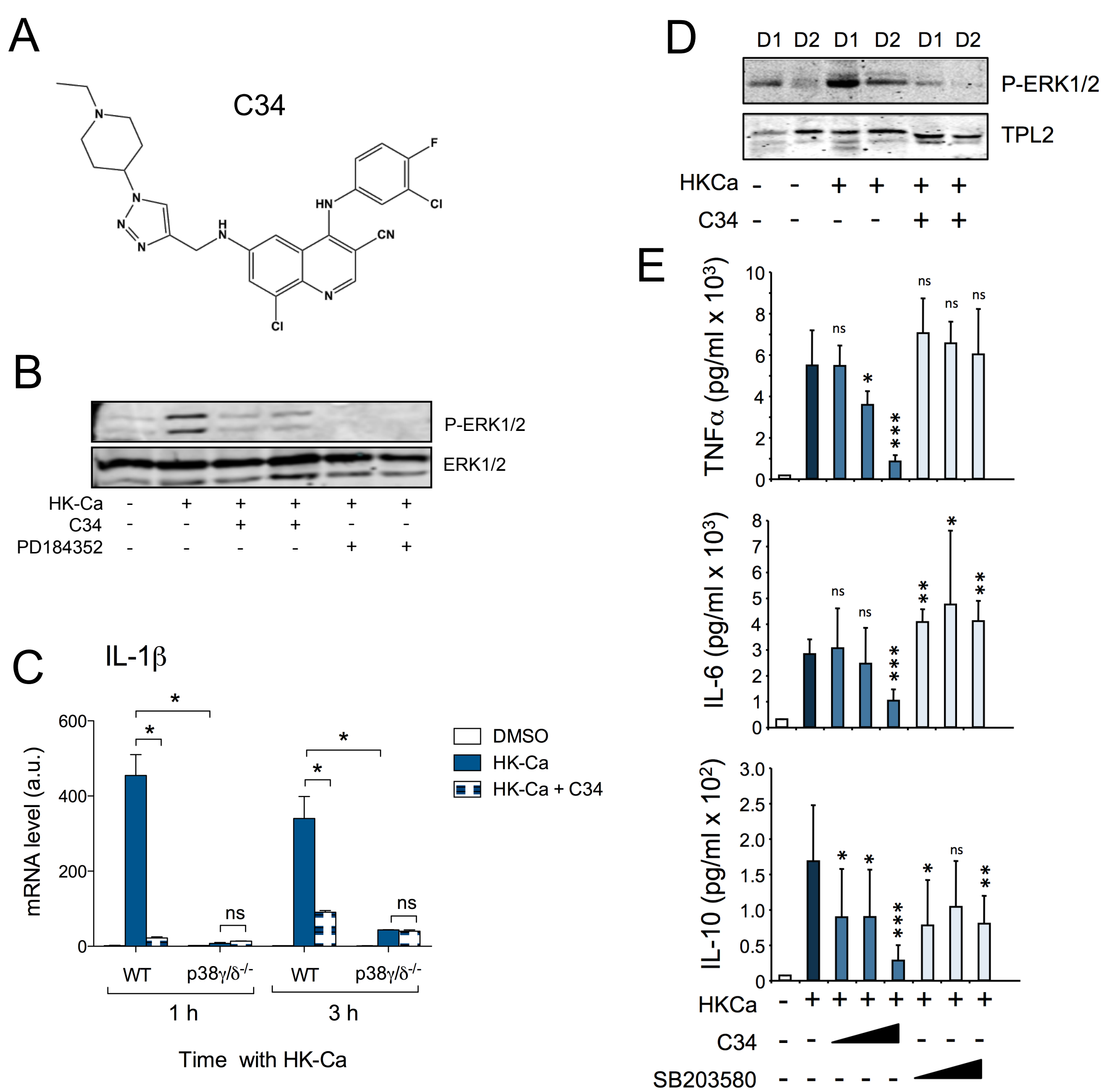
A
C34
B
P-ERK1/2
ERK1/2
HK-Ca
C34
PD184352
C
IL-1β
mRNA level (a.u.)
DMSO
HK-Ca
HK-Ca + C34
WT
p38γ/δ-/-
1 h
3 h
Time with HK-Ca
D
D1 D2 D1 D2 D1 D2
P-ERK1/2
TPL2
HKCa
C34
E
TNFα (pg/ml x 10^3)
IL-6 (pg/ml x 10^3)
IL-10 (pg/ml x 10^2)
HKCa
C34
SB203580

Figure S2

Fig S2. TPL2 regulates the activation of macrophages mediated by *C. albicans*.

(**A**) Chemical structure of the TPL2 inhibitor, the compound C34.

(**B**) WT BMDM were incubated for 1 h with DMSO or with 5 μM C34 or 2 μM PD184352, and then stimulated for 1 h with 1 x 10^6 CFU/ml HK-Ca. Cell lysates were immunoblotted with the indicated antibodies. Representative immunoblots are shown.

(**C**) BMDM were incubated for 1 h with or without 5 μM C34, and then exposed for 1 h to 1 x 10^6 CFU/ml HK-Ca. Relative mRNA expression for IL-1β was determined by qPCR. Results were normalized and fold induction calculated. Data show mean ± SEM from one representative experiment of two in triplicate, with similar results. ns, not significant, * $p \leq 0.05$ relative to WT BMDM exposed to HK-Ca, in each time.

(**D**) TPL2 modulates cytokine production in response to *C. albicans* in human monocytes. Monocytes from healthy donors (D1, D2) were incubated for 1 h with DMSO or 5 μM C34, and then stimulated for 1 h with 1 x 10^6 CFU/ml HK-Ca. Cell lysates (30μg) were immunoblotted with the indicated antibodies. Representative blots are shown.

(**E**) Monocytes were incubated for 1 h in the absence (DMSO) or the presence of 0.5μM, 1.5μM and 5μM C34 or SB203580 and then stimulated as in (D). Cytokine production was measured in the supernatants by ELISA. Representative data from three independent experiments are shown. Data show mean ± SEM (n= 4 donors). ns, not significant; *$p \leq 0.05$; **$p \leq 0.01$, ***$p \leq 0.001$, relative to stimulated cells in the absence of inhibitor.

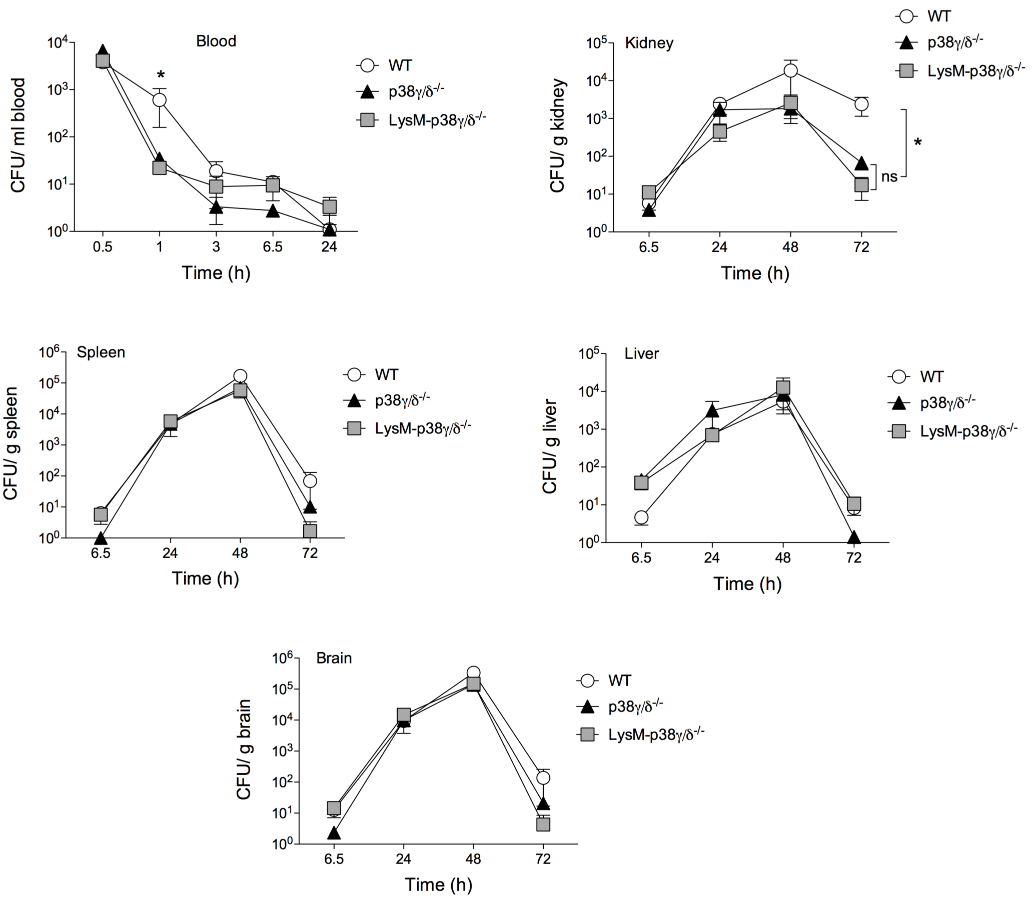

Figure S3

Fig S3. Fungal burden in different organs after systemic *C. albicans* infection.

WT, p38γ/δ$^{-/-}$ and LysM-p38γ/δ$^{-/-}$ mice were infected with 1x10^{5} CFU of *C. albicans* and fungal burden was analysed in the indicated organs at the indicated times. Data are expressed as mean ± SEM (*n* = 6 in kidney and blood; *n* = 3 in spleen, liver and brain). ns, not significant, **p* ≤ 0.05 relative to WT mice.

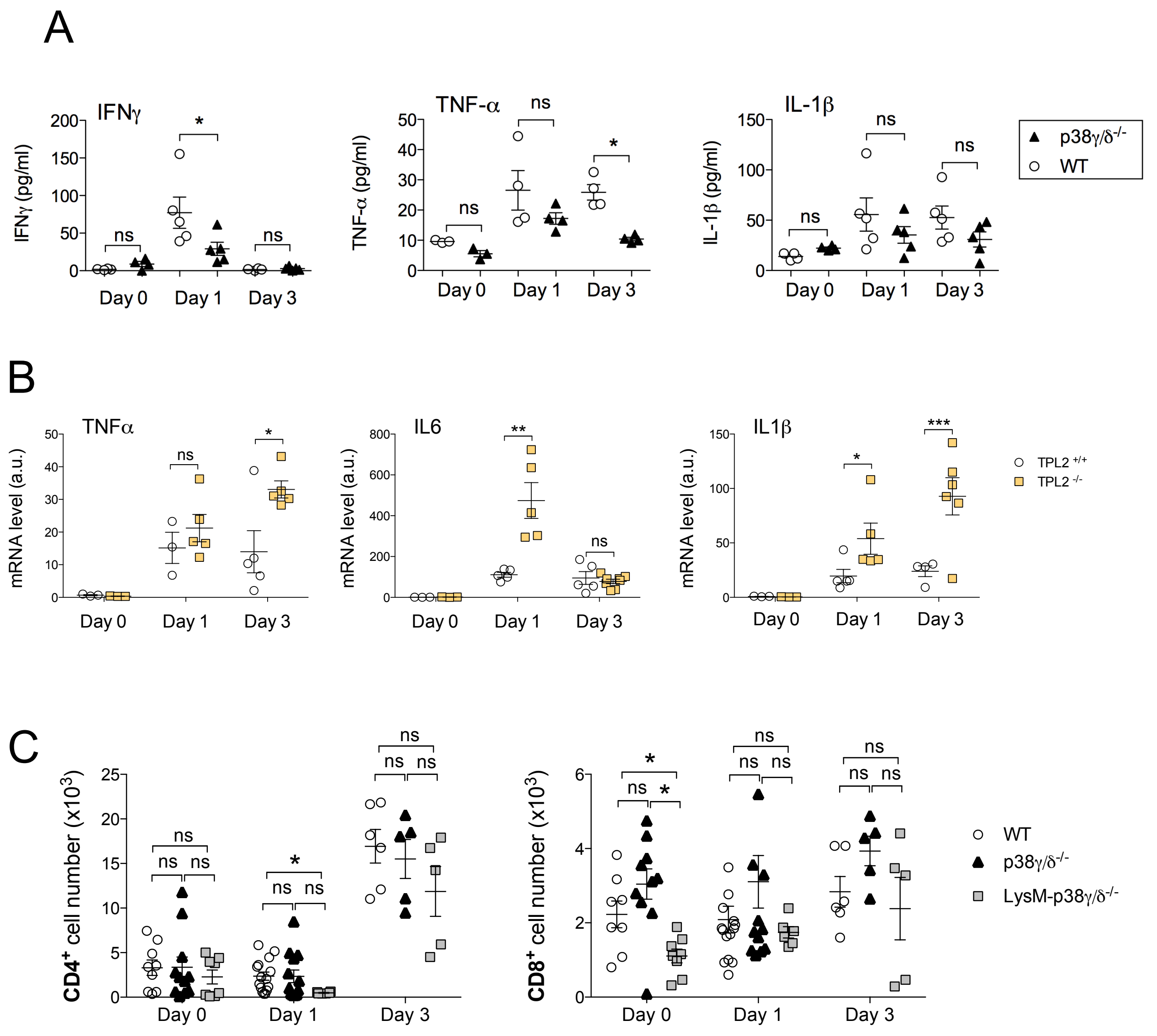
A
IFNγ
IFNγ (pg/ml)
TNF-α
TNF-α (pg/ml)
IL-1β
IL-1β (pg/ml)
p38γ/δ-/-
WT
Day 0
Day 1
Day 3
ns
*
B
TNFα
IL6
IL1β
mRNA level (a.u.)
TPL2 +/+
TPL2 -/-
**
***
C
CD4+ cell number (x10³)
CD8+ cell number (x10³)
WT
p38γ/δ-/-
LysM-p38γ/δ-/-

Figure S4

Fig S4. Cytokine production and cell recruitment in response to *C. albicans* infection.

(**A**) Serum from WT and p38γ/δ$^{-/-}$ mice was collected 0, 1 and 3 days after infection with 1 x 10^5 CFU *C. albicans* and TNFα, IFNγ and IL-1β measured in a luminex cytokine assay. Each symbol represents an individual mouse. ns, not significant and **p* ≤ 0.05 relative to WT mouse serum.

(**B**) TPL2$^{+/+}$ and TPL2$^{-/-}$ mice were intravenously infected with 1 x 10^5 CFU *C. albicans* and at days 1 and 3 post-infection, relative TNFα, IL6 and IL-1β mRNA expression in the kidney was determined by qPCR and normalised to β-actin mRNA. Each symbol represents an individual mouse. ns, not significant, **p* ≤0.05, ***p* ≤0.01, ****p* ≤0.001 relative to TPL2$^{+/+}$ mice.

(**C**) Kidney cells from 0-, 1- and 3-day *C. albicans*-infected WT, p38γ/δ$^{-/-}$ and LysM-p38γ/δ$^{-/-}$ mice were stained with anti-CD45, -CD4 and -CD8 antibodies. CD45$^+$ cells were gated and absolute numbers of CD4$^+$ and CD8$^+$ cells were calculated. Each symbol represents an individual mouse. Figure shows mean ± SEM, (ns) not significant, **p* ≤0.05; ***p* ≤0.01, ****p* ≤0.001, relative to WT mice, at each time.

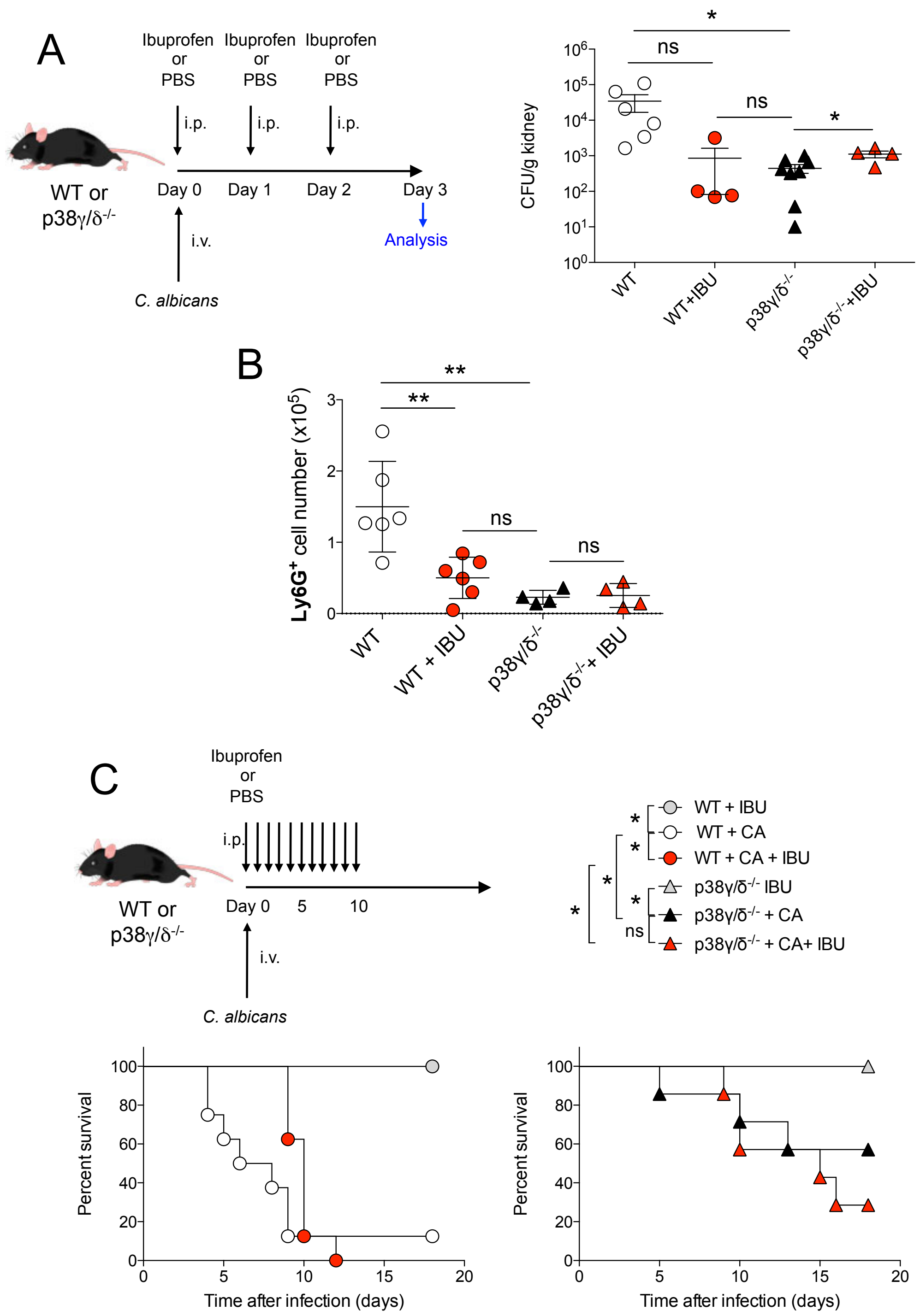
A
Ibuprofen or PBS
i.p.
WT or p38γ/δ-/-
Day 0
Day 1
Day 2
Day 3
Analysis
i.v.
C. albicans
CFU/g kidney
*
ns
ns
*
WT
WT+IBU
p38γ/δ-/-
p38γ/δ-/-+IBU
B
Ly6G+ cell number (x105)
**
**
ns
ns
WT
WT + IBU
p38γ/δ-/-
p38γ/δ-/- + IBU
C
Ibuprofen or PBS
i.p.
WT or p38γ/δ-/-
Day 0 5 10
i.v.
C. albicans
WT + IBU
WT + CA
WT + CA + IBU
p38γ/δ-/- IBU
p38γ/δ-/- + CA
p38γ/δ-/- + CA+ IBU
*
*
*
*
*
ns
Percent survival
Time after infection (days)

Figure S5

Fig S5. Effect of Ibuprofen in *C. albicans* infected mice.

(**A**) WT and p38γ/δ$^{-/-}$ mice were intravenously injected with 1 x 10^5 CFU of *C. albicans* and treated with 30 mg per kg body weight per day of ibuprofen sodium salt from SIGMA (IBU) or with the same volume of the vehicle PBS. Kidney fungal load was determined 3 days after infection. ns, not significant, *p ≤0.05. Each symbol represents an individual mouse.

(**B**) Neutrophil infiltration in the kidney of infected WT and p38γ/δ$^{-/-}$ mice, treated with ibuprofen (IBU) as in (A) was determined by flow cytometry. Kidney cells from 3-day *C. albicans*-treated WT and p38γ/δ$^{-/-}$ treated or not with ibuprofen were stained with anti-CD45 and -Ly6G antibodies and positive cells analysed by flow cytometry. Each symbol represents an individual mouse. Figure shows mean ± SEM, ns not significant; **p ≤0.01.

(**C**) WT and p38γ/δ$^{-/-}$ mice were infected with 1 x 10^5 CFU *C. albicans* and treated with ibuprofen as in (A) for 10 days (WT + CA+ IBU (n = 8); p38γ/δ$^{-/-}$ + CA + IBU (n = 7)). Control groups of WT and p38γ/δ$^{-/-}$ mice treated with 30 mg per kg body weight per day of ibuprofen were included to check its toxicity (WT + IBU (n = 6); p38γ/δ$^{-/-}$ + IBU (n = 6)). Control groups of WT and p38γ/δ$^{-/-}$ mice infected with *C. albicans* were also included for comparison (WT + CA (n = 8); p38γ/δ$^{-/-}$ + CA (n = 7)). Survival was monitored as indicated. Data are presented as a Kaplan-Maier plot. ns, not significant, *p ≤0.05; **p ≤0.01.

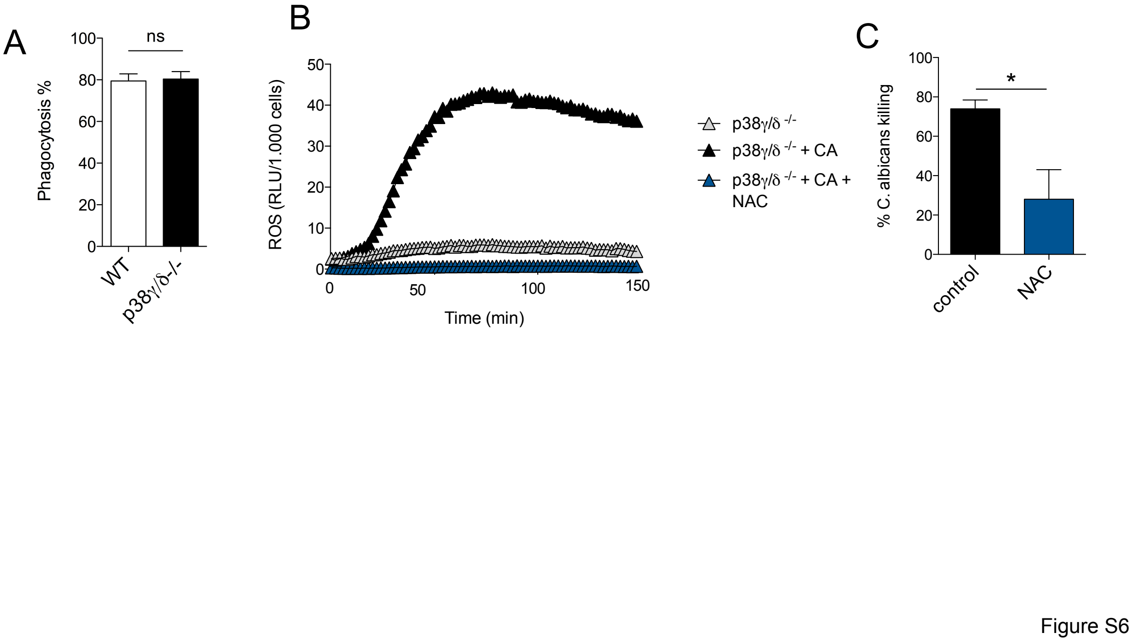

Fig S6. Effect of the antioxidant agent NAC in *C. albicans* infection.

(**A**) *C. albicans* phagocytosis by BMDM. The results are represented as percentage of phagocytosed fungus. The *C.albicans*/BMDM ratio was 1:10. Values are mean ± SEM (n = 6); ns, not significant.

(**B**) ROS production in p38γ/δ$^{-/-}$ BMDM treated with 1.5 x 10^6 CFU/ml HK-Ca in the presence (p38γ/δ$^{-/-}$ + CA + NAC) or absence (p38γ/δ$^{-/-}$ + CA) of 5 mM NAC. Control without *C. albicans* infection was also included in the experiments. Experiments were performed in triplicate. RLU, relative light unit.

(**C**) *C. albicans* killing by p38γ/δ$^{-/-}$ BMDM in the presence of NAC. p38γ/δ$^{-/-}$ BMDM were incubated with 5 mM NAC or the same volume of PBS for 1 h before infecting with *C. albicans*. The *C. albicans*/BMDM ratio was 1:10. The results are represented as percentage of killing (the percentage of killed fungal cells among the phagocytosed fungus). Values are mean ± SEM (n = 4); * $p \leq 0.05$ relative to control cells.

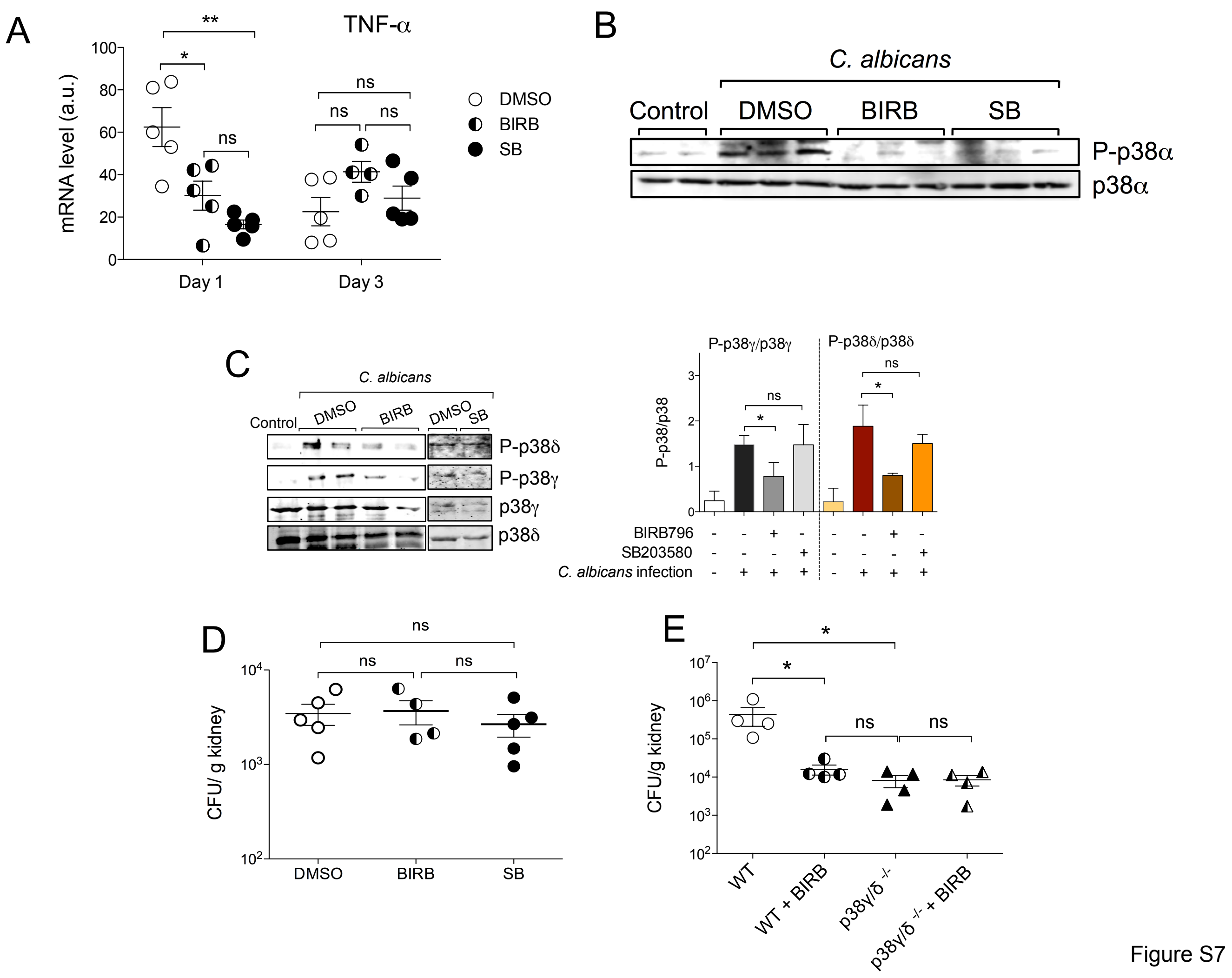

Fig S7. Fungal burden in the kidney of *C. albicans* infected mice treated with BIRB796 and SB203580.

(**A**) WT mice were intravenously infected with 1 x 10^5 CFU *C. albicans* and treated with 10 mg per kg body weight per day of BIRB796 (BIRB) or SB203580 (SB), or with the same volume of the vehicle DMSO. At days 1 and 3 post-infection, relative TNFα mRNA expression in the kidney was determined by qPCR and normalised to β-actin mRNA. Each symbol represents an individual mouse. ns, not significant, *p ≤0.05, **p ≤0.01 relative to control mice.

(**B**) WT mice were treated as in (A). 24 h later, protein extracts obtained from two to three mouse kidneys were immunoblotted with anti-P-p38 (P-p38α) and total p38α. Representative blots from two independent experiments are shown.

(**C**) Endogenous p38δ and p38γ were immunoprecipitated from kidney extracts (8 mg) 24 h after *C. albicans* infection and inhibitor treatment. Pellets were immunoblotted with anti-phospho-p38 (that recognises P-p38δ, P-p38γ), anti-p38γ or anti-p38δ antibodies. Representative blots are shown. Bands from immunoblots were quantified using the Odyssey infrared imaging system and represented as P-p38 density/p38 (P-p38γ/p38γ or P-p38δ/p38δ). Data show mean ± SD (*n* = 2-4).

(**D**) Kidney fungal load was determined 1 day after infection. ns, not significant.

(**E**) WT and p38γ/$\delta^{-/-}$ mice were intravenously injected with 1 x10^5 CFU of *C. albicans* and treated with 10 mg per kg body weight per day of BIRB796 or with the same volume of the vehicle DMSO. Kidney fungal load was determined 3 days after infection. ns, not significant, *$p \leq 0.05$.

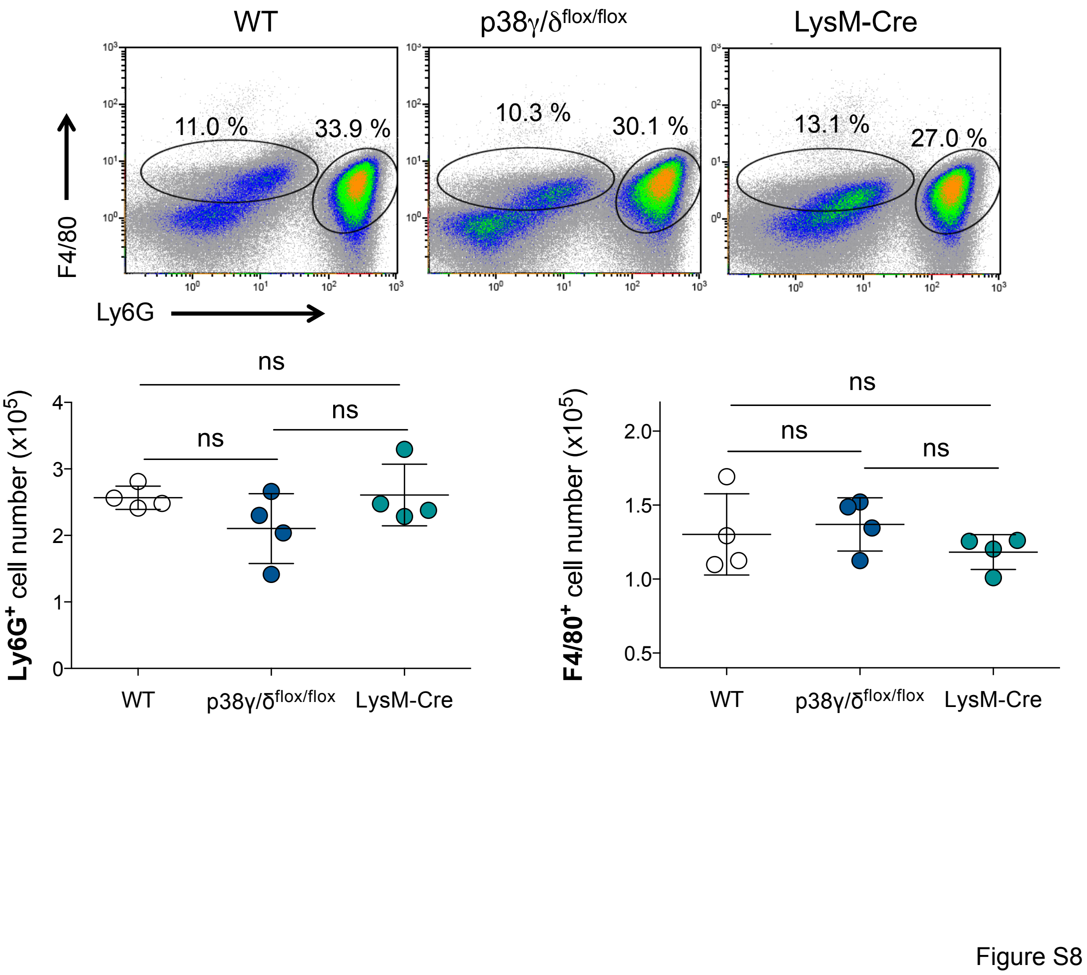

Fig S8. Cell recruitment in the peritoneum of control mice after *C. albicans* infection.

Mice of the indicated genotypes were intraperitoneally infected with 5 x 10^6 CFU *C. albicans* and at day 1 post-infection, peritoneal cells were stained with anti-CD45, -Ly6G and -F4/80 antibodies and positive cells analysed by flow cytometry. $CD45^+$ cells were gated and $-F4/80^+$ and $-Ly6G^+$ cells analysed by flow cytometry. Representative profiles are shown. Each symbol represents an individual mouse. Figure shows mean ± SEM, ns not significant.
