# Supplementary material for: Myeloid cell deficiency of p38γ/p38δ protects against candidiasis and regulates antifungal immunity
Source: EMBO Mol Med. 2018 Apr 16;10(5):e8485. doi: 10.15252/emmm.201708485 (PMC5938613; doi:10.15252/emmm.201708485)

Source Data- Appendix Figure S1A

Imiquimod

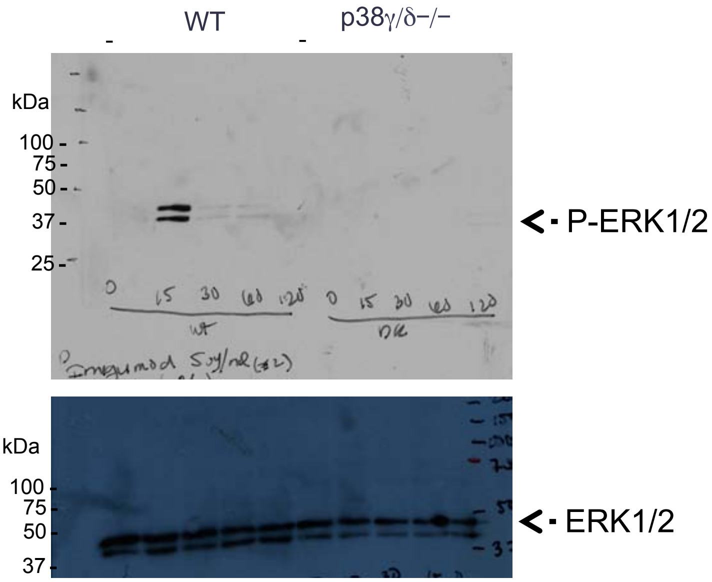

ODN

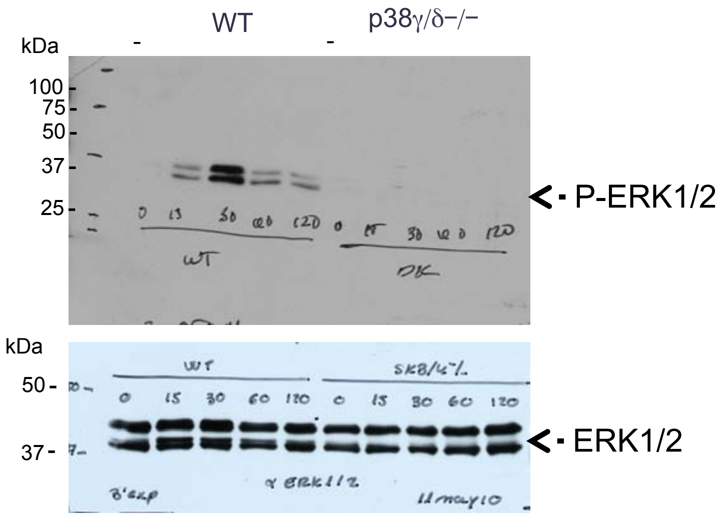

Source Data- Appendix Figure S1B

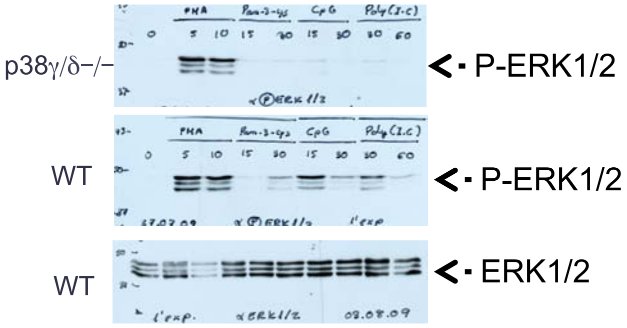

Source Data- Appendix Figure S1C

Imiquimod

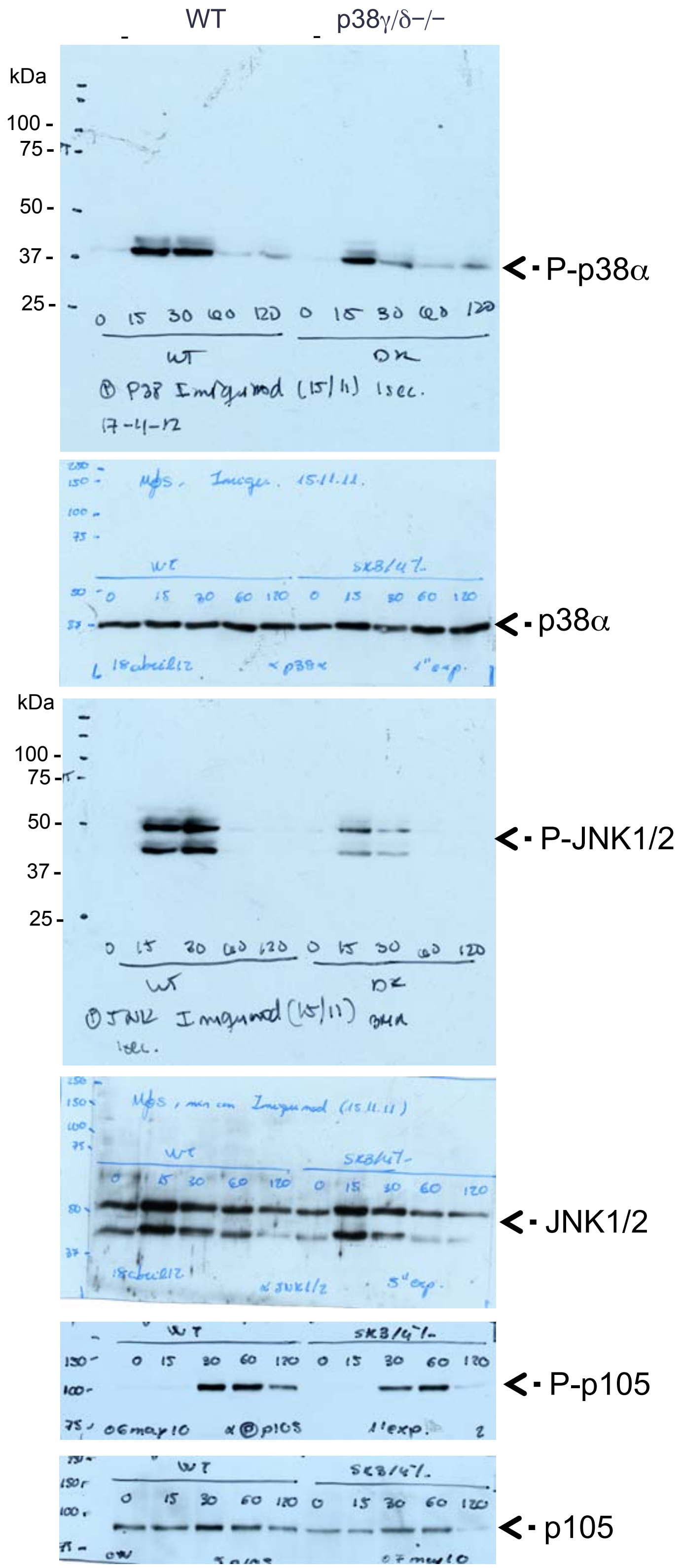

Source Data- Appendix Figure S1C

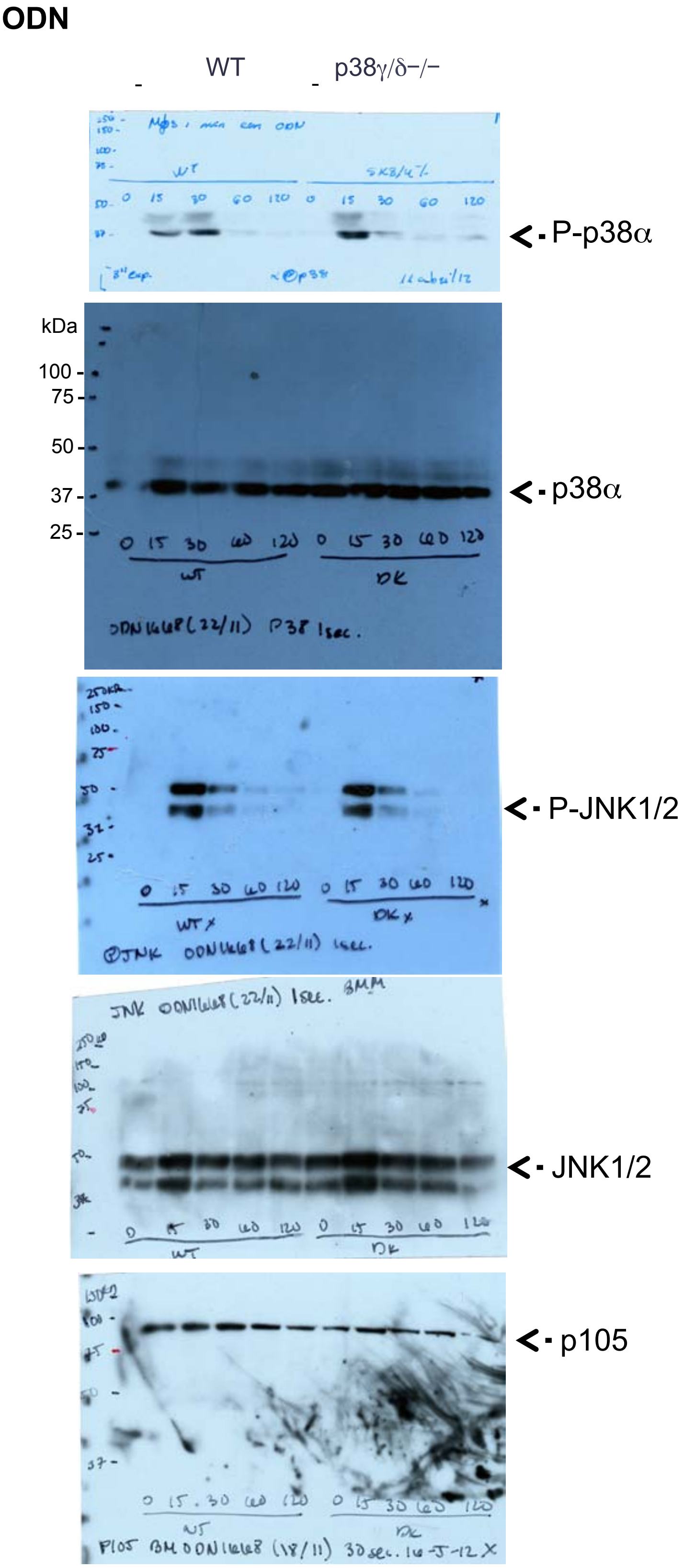

Source Data- Appendix Figure S1C

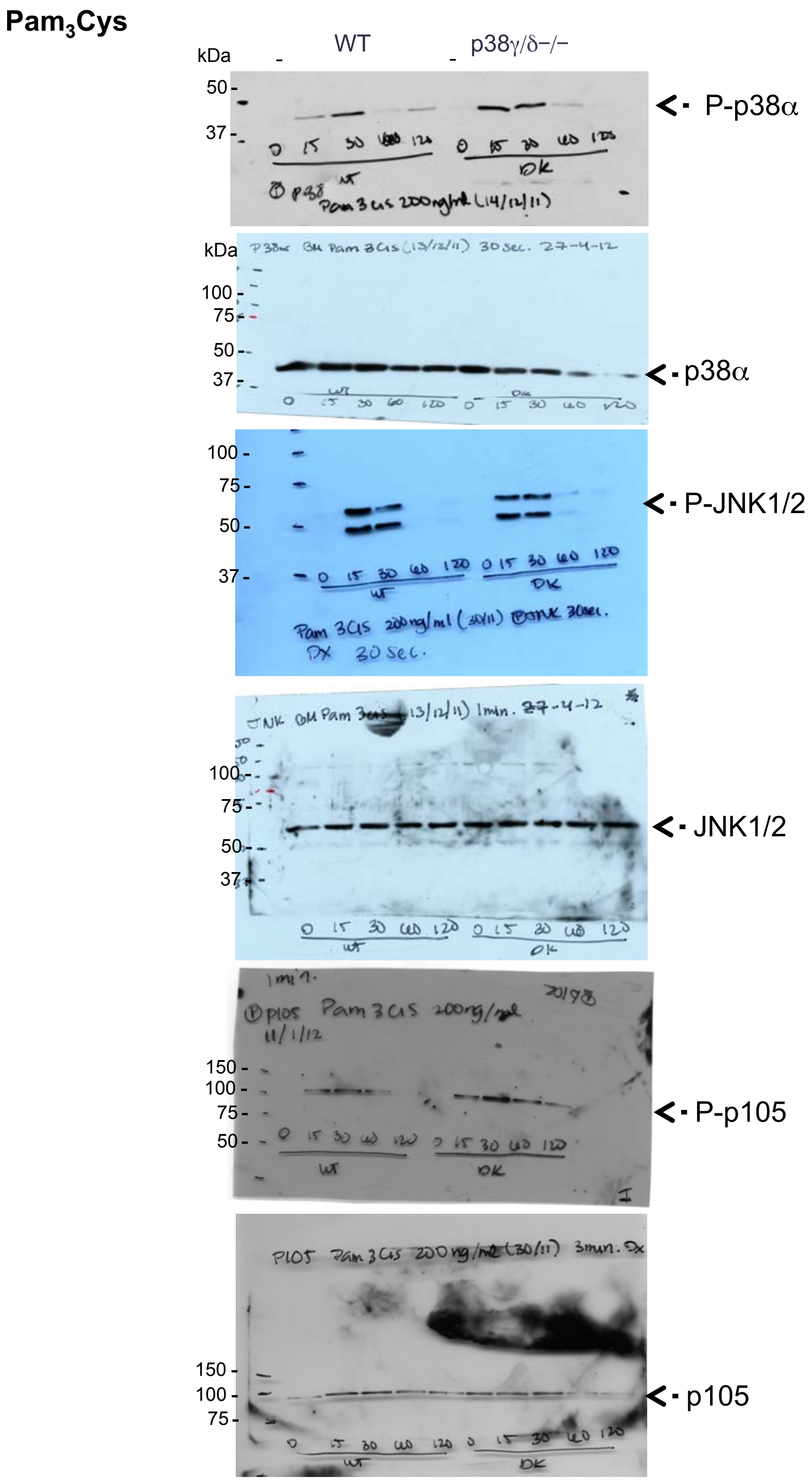

Source Data- Appendix Figure S1D

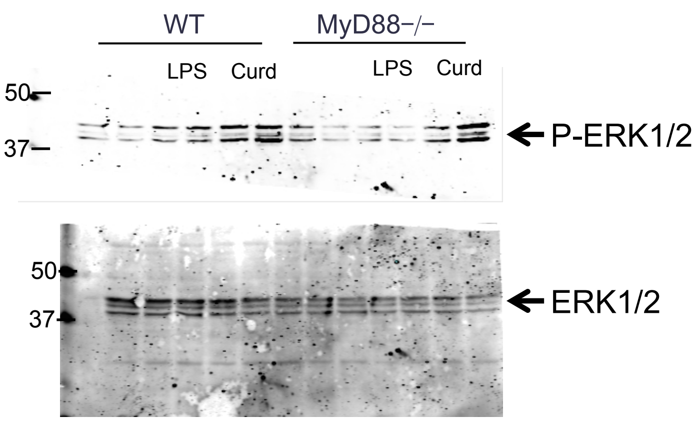

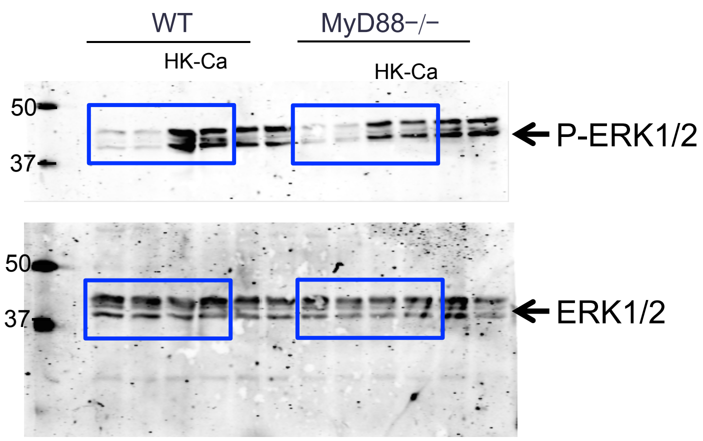

Source Data- Appendix Figure S1E

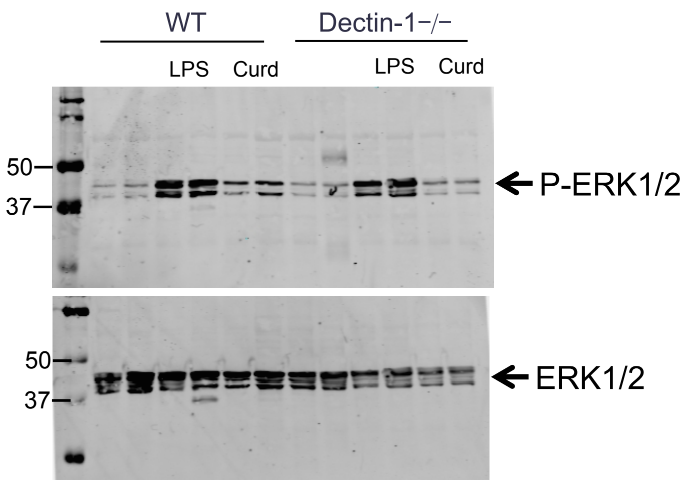

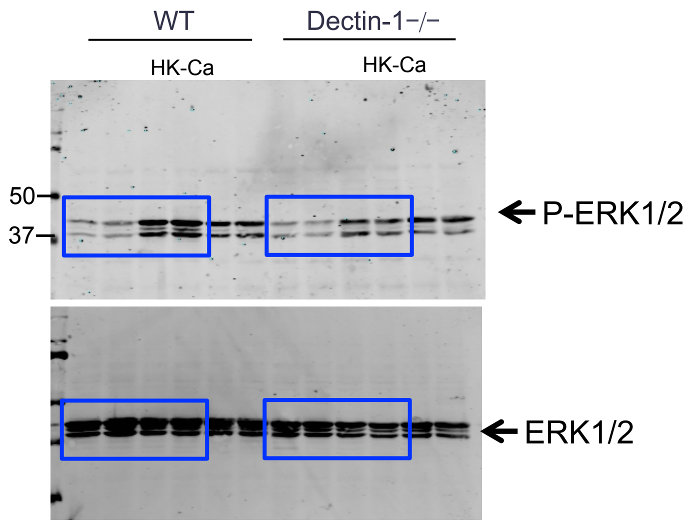

Source Data- Appendix Figure S1F

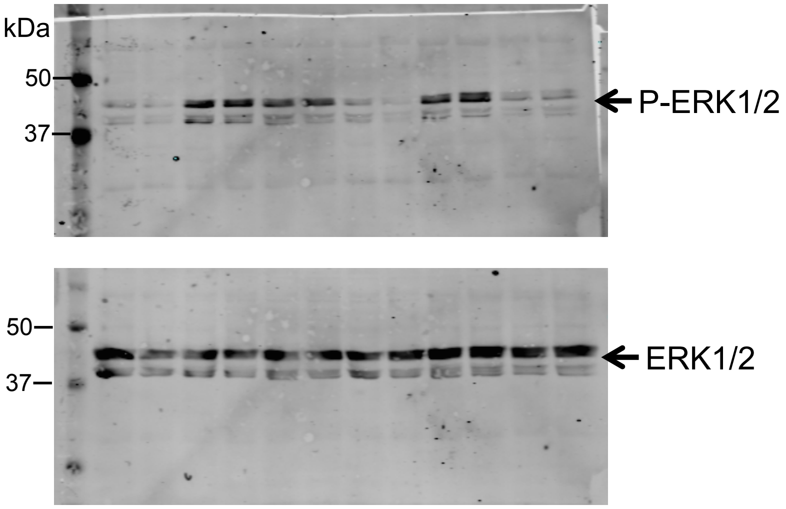

Supplement: Supplementary file 2 — Source Data for Appendix [file EMMM-10-e8485-s004.zip › Source_data_S1.pdf]
