# Supplementary figures and images for: Myeloid cell deficiency of p38γ/p38δ protects against candidiasis and regulates antifungal immunity
Source: EMBO Mol Med. 2018 Apr 16;10(5):e8485. doi: 10.15252/emmm.201708485 (PMC5938613; doi:10.15252/emmm.201708485)

## Source Data- Appendix Figure S2D

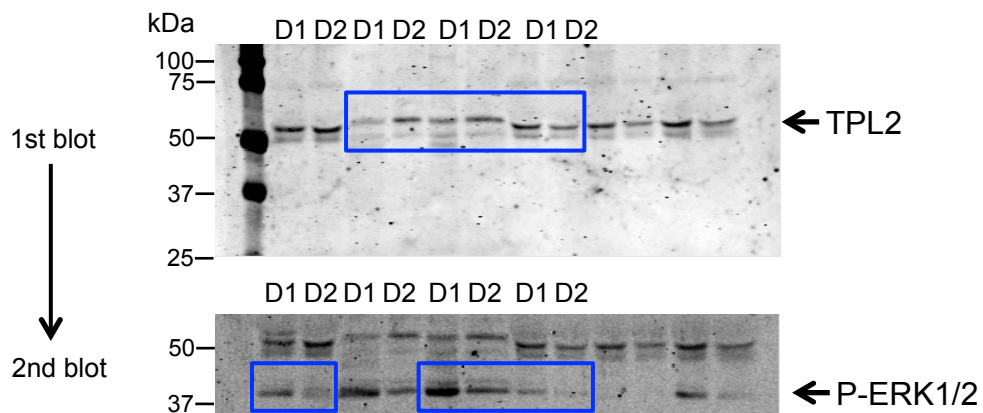

Supplement: Supplementary file 2 — Source Data for Appendix [file EMMM-10-e8485-s004.zip › Source_data_S2.pdf]

Source Data-Appendix Figure S7B

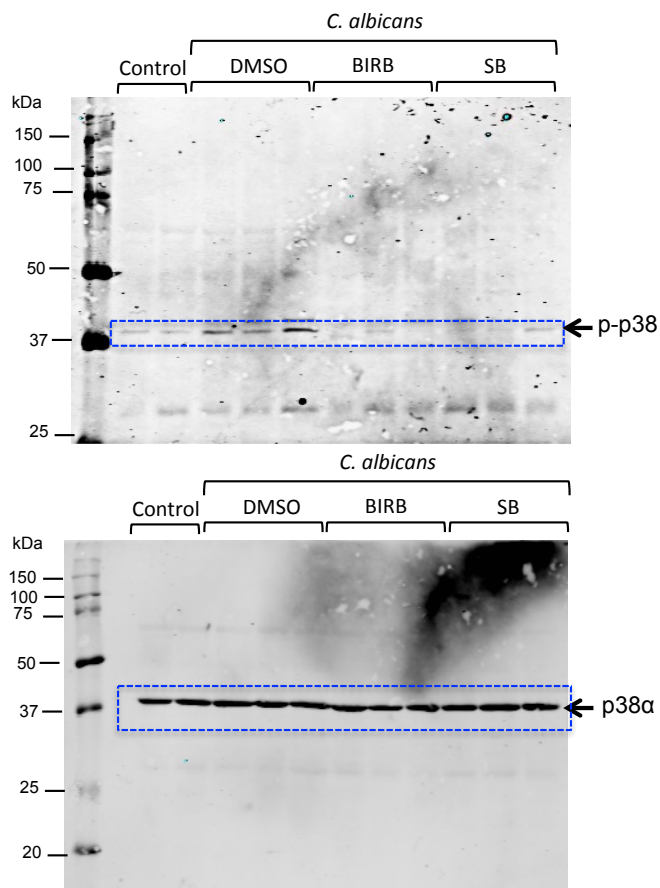

Source Data-Appendix Figure S7C

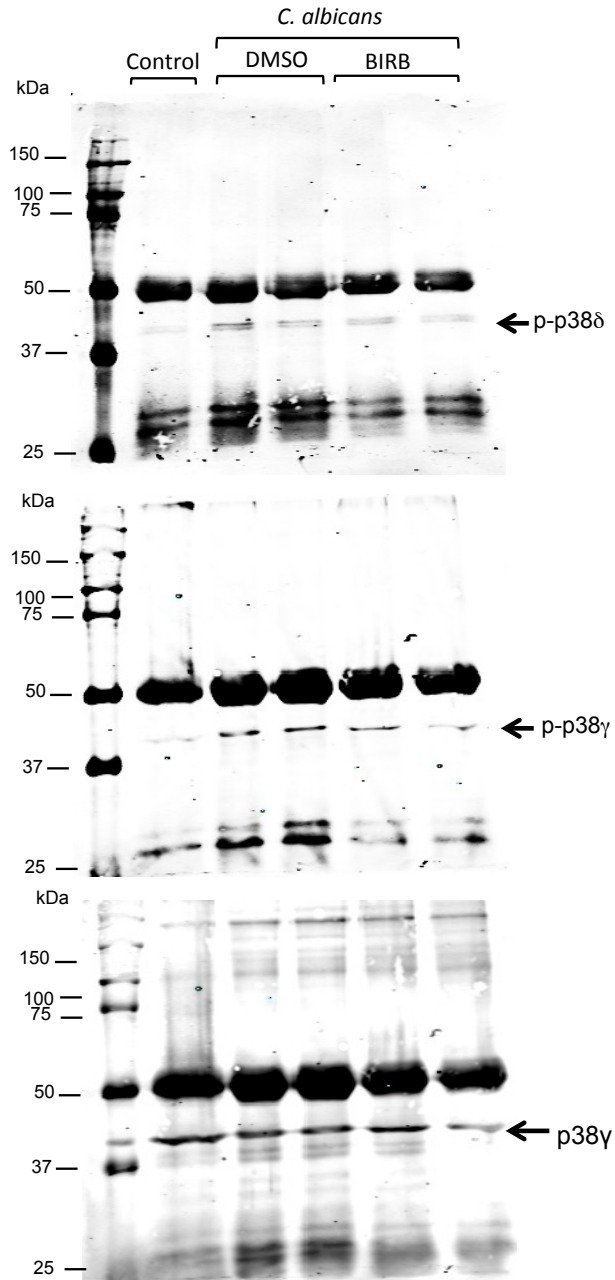

Supplement: Supplementary file 2 — Source Data for Appendix [file EMMM-10-e8485-s004.zip › Source_data_S7.pdf]

Source Data- Figure 1C

Pam<sub>3</sub>Cys

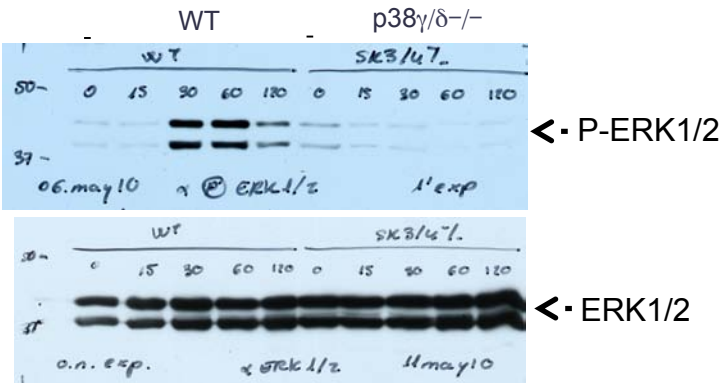

LPS

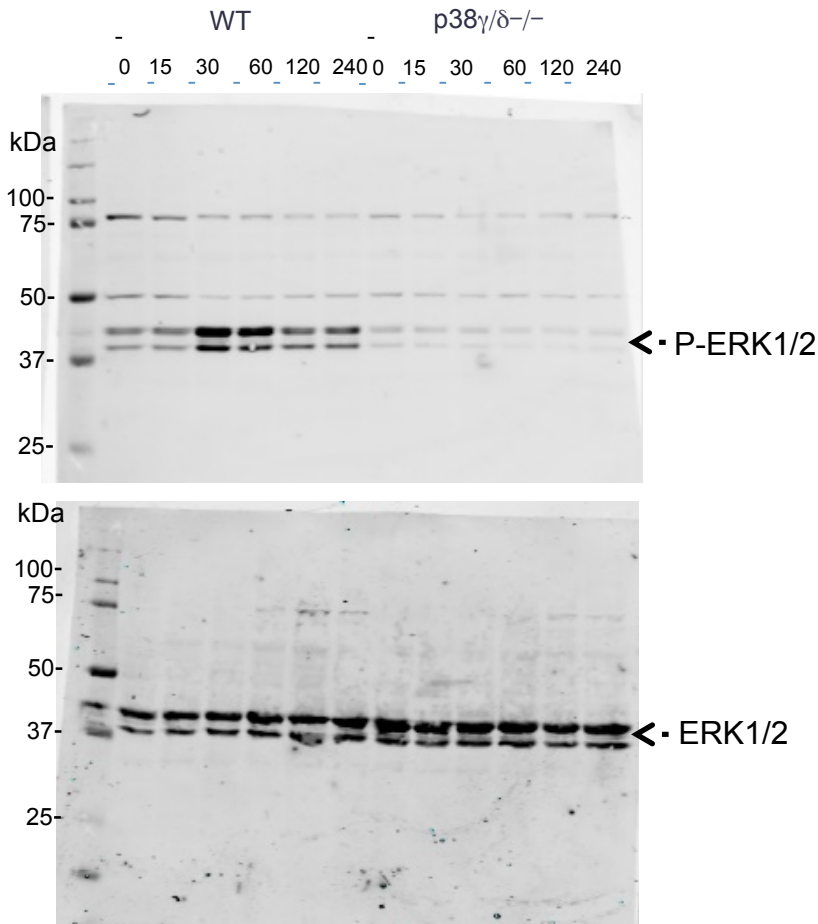

Source Data- Figure 1E

Curdlan

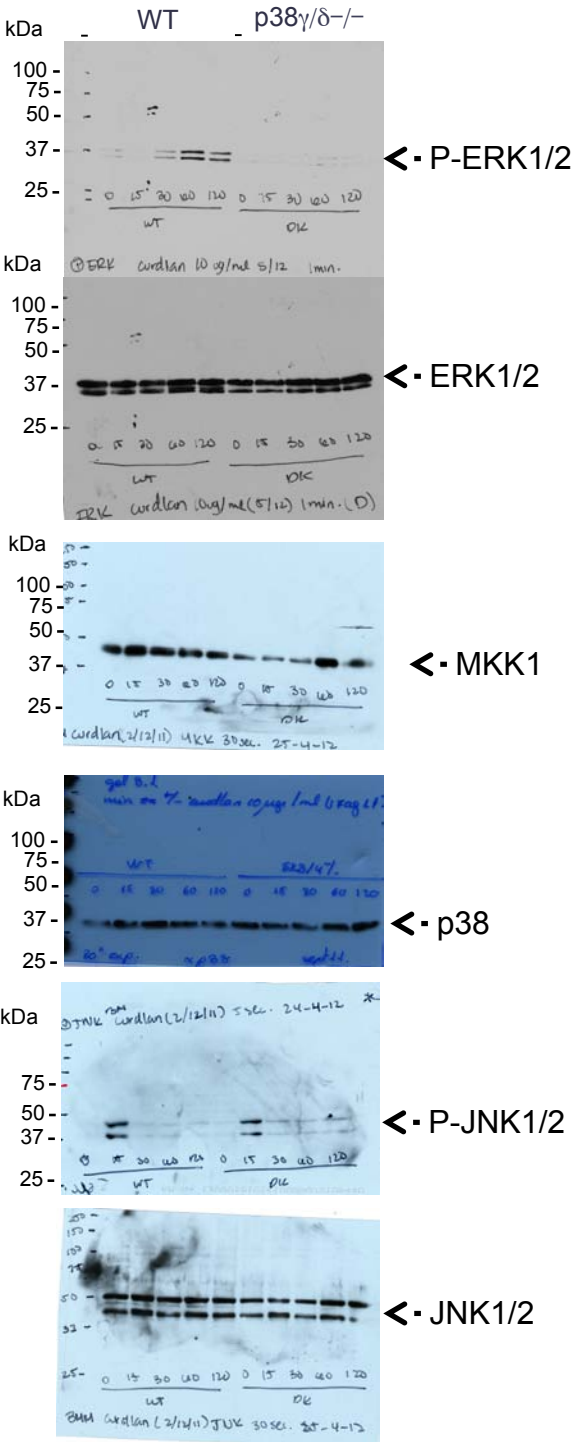

Supplement: Supplementary file 4 — Source Data for Figure 1 [file EMMM-10-e8485-s002.pdf]

Source Data- Figure 2A

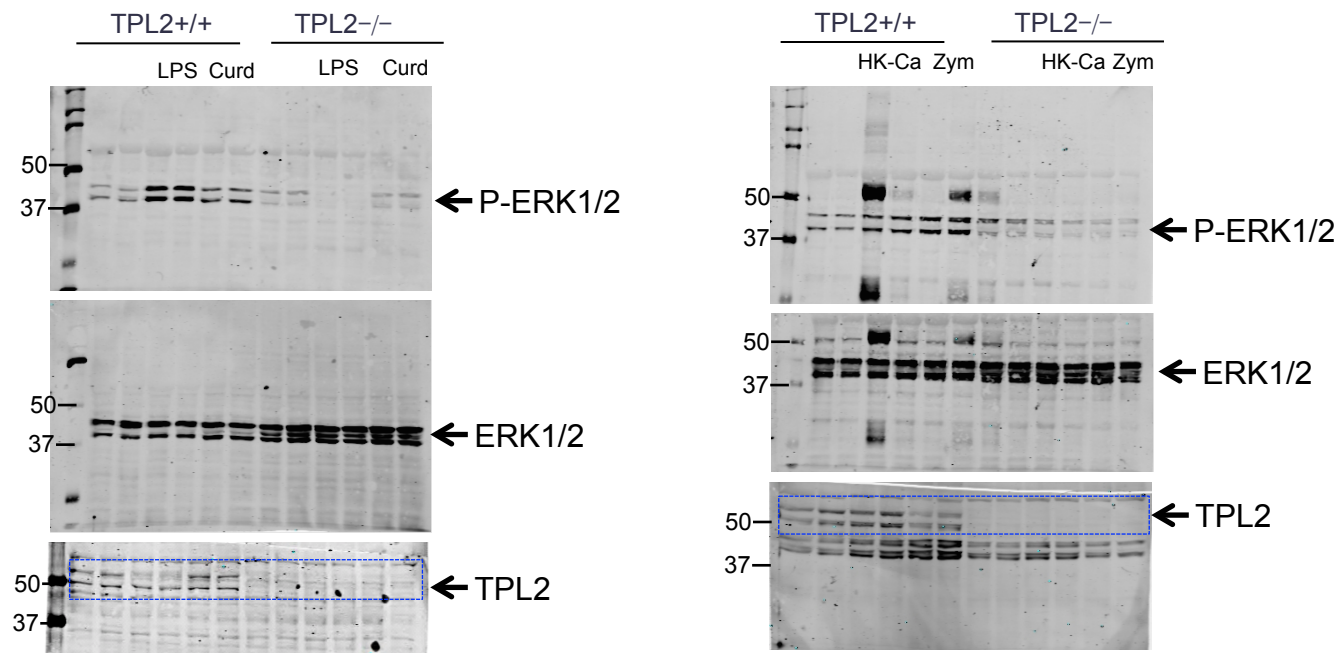

Source Data- Figure 2B

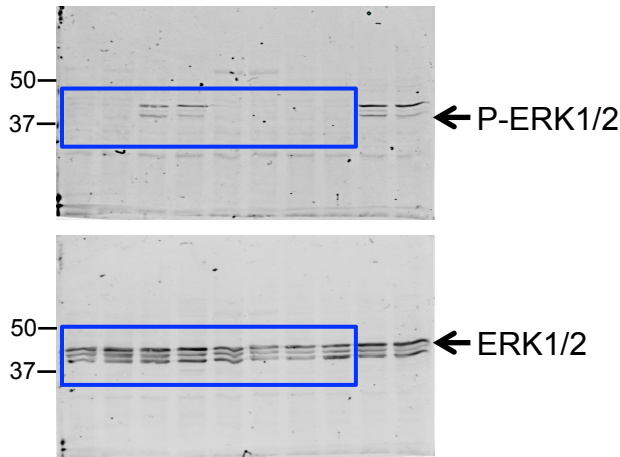

Source Data- Figure 2D

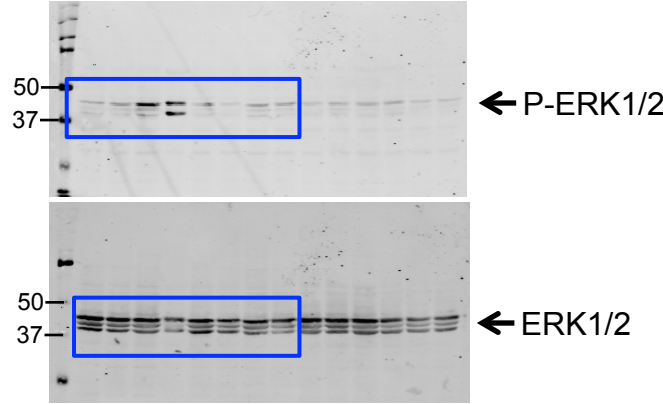

Source Data- Figure 2E

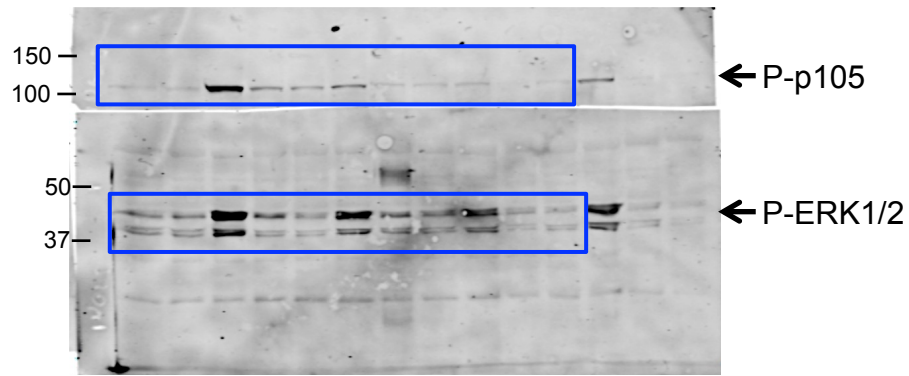

Supplement: Supplementary file 5 — Source Data for Figure 2 [file EMMM-10-e8485-s003.pdf]
